# SPL7013 Gel (VivaGel®) Retains Potent HIV-1 and HSV-2 Inhibitory Activity following Vaginal Administration in Humans

**DOI:** 10.1371/journal.pone.0024095

**Published:** 2011-09-15

**Authors:** Clare F. Price, David Tyssen, Secondo Sonza, Ashley Davie, Sonya Evans, Gareth R. Lewis, Shirley Xia, Tim Spelman, Peter Hodsman, Thomas R. Moench, Andrew Humberstone, Jeremy R.A. Paull, Gilda Tachedjian

**Affiliations:** 1 Starpharma Pty Ltd, Melbourne, Victoria, Australia; 2 Centre for Virology, Burnet Institute, Melbourne, Victoria, Australia; 3 Department of Microbiology, Monash University, Clayton, Victoria, Australia; 4 Centre for Population Health, Burnet Institute, Melbourne, Victoria, Australia; 5 Nucleus Network, Melbourne, Victoria, Australia; 6 ReProtect, Inc., Baltimore, Maryland, United States of America; 7 Department of Medicine, Monash University, Melbourne, Victoria, Australia; University of Alabama, United States of America

## Abstract

SPL7013 Gel (VivaGel^®^) is a microbicide in development for prevention of HIV and HSV. This clinical study assessed retention and duration of antiviral activity following vaginal administration of 3% SPL7013 Gel in healthy women. Participants received 5 single doses of product with ≥5 days between doses. A cervicovaginal fluid (CVF) sample was collected using a SoftCup™ pre-dose, and immediately, or 1, 3, 12 or 24 h post-dose. HIV-1 and HSV-2 antiviral activities of CVF samples were determined in cell culture assays. Antiviral activity in the presence of seminal plasma was also tested. Mass and concentration of SPL7013 in CVF samples was determined. Safety was assessed by reporting of adverse events. Statistical analysis was performed using the Wilcoxon signed-rank test with Bonferroni adjustment; p≤0.003 was significant. Eleven participants completed the study. Inhibition of HIV-1 and HSV-2 by pre-dose CVF samples was negligible. CVF samples obtained immediately after dosing almost completely inhibited (median, interquartile range) HIV-1 [96% (95,97)] and HSV-2 [86% (85,94)], and activity was maintained in all women at 3 h (HIV-1 [96% (95,98), p = 0.9]; HSV-2 [94% (91,97), p = 0.005]). At 24 h, >90% of initial HIV-1 and HSV-2 inhibition was maintained in 6/11 women. SPL7013 was recovered in CVF samples obtained at baseline (46% of 105 mg dose). At 3 and 24 h, 22 mg and 4 mg SPL7013, respectively, were recovered. More than 70% inhibition of HIV-1 and HSV-2 was observed if there was >0.5 mg SPL7013 in CVF samples. High levels of antiviral activity were retained in the presence of seminal plasma. VivaGel was well tolerated with no signs or symptoms of vaginal, vulvar or cervical irritation reported. Potent antiviral activity was observed against HIV-1 and HSV-2 immediately following vaginal administration of VivaGel, with activity maintained for at least 3 h post-dose. The data provide evidence of antiviral activity in a clinical setting, and suggest VivaGel could be administered up to 3 h before coitus.

**Trial Registration:**

The study is registered at ClinicalTrials.gov under identifier: NCT00740584

## Introduction

The global human immunodeficiency virus (HIV) epidemic continues, with an estimated 2.7 million people newly infected with HIV and 2.0 million dying of acquired immune deficiency syndrome (AIDS) in 2008 [1]. Women account for half of all people living with HIV worldwide, increasing to nearly 60% of HIV infections in sub-Saharan Africa [1]. Vaginally applied microbicide agents offer a potential strategy for prevention of the sexual transmission of HIV or other sexually transmitted infections (STIs) that can be initiated by women. Microbicide classes include non-specific surfactants or detergents and acid buffering agents, moderately specific macromolecular anionic polymers that block HIV and other STIs, and HIV specific drugs that inhibit viral entry and reverse transcription [2]. A proof of concept study demonstrating that a vaginal topical microbicide gel containing 1% tenofovir can protect women against HIV acquisition with 39% efficacy compared to placebo gel provides credence to the development of topical microbicides for HIV prevention [3].

SPL7013 Gel (VivaGel®) is a vaginal microbicide being developed by Starpharma Pty Ltd (Melbourne, Australia) for the prevention of HIV and herpes simplex virus (HSV) infections [4]. The active ingredient, SPL7013, is a dendrimer comprising a divalent benzhydrylamine (BHA) core, four generations of lysine branches with the outermost branches capped with a total of 32 naphthalene disulfonic acid groups that impart hydrophobicity and a high anionic charge to the dendrimer surface [5]. SPL7013 has been formulated in a mucoadhesive Carbopol^®^-based aqueous gel for use as a topical vaginal microbicide [6], [7].

SPL7013 has broad-spectrum activity against HIV-1 clades and HIV-2 *in vitro* [5], [8], [9] by inhibiting viral attachment and entry [5], [9], [10]. In contrast to most linear polyanion microbicides, SPL7013 demonstrates similar potency against HIV-1 that utilize the CXCR4 and CCR5 chemokine receptors [5], the latter accounting for the majority of HIV transmissions [11]. SPL7013 is active against HIV in cell culture assays in the presence of high concentrations of serum and cervicovaginal secretions [5], and retains HIV inhibitory activity when diluted 30-fold in seminal plasma [9]. SPL7013 also inhibits HIV-1 replication in colorectal and cervical explant systems against a CCR5 using strain [12], [13] and does not specifically enhance HIV-1 replication *in vitro* [14]. SPL7013 has low toxicity in cervical and colorectal epithelial cell lines and lacks the ability to disrupt intracellular tight junctions of polarized epithelial cells [8].

In a macaque model, SPL7013 Gel was protective against vaginal challenge with a CXCR4 using simian humanised immunodeficiency virus (SHIV_89.6P_) in a dose-related manner and inhibits replication of the CCR5 using SHIV_162P3_ strain in macaque and human peripheral blood mononuclear cells (PBMCs) [15]. Activity against HSV-1 and HSV-2 has been demonstrated *in vitro* [5], [10], [16], and against HSV-2 infection in mice and guinea pigs [5], [16]. A contraceptive effect of SPL7013 Gel has also been observed in rabbits [4]. SPL7013 Gel is not toxic as demonstrated in animal models [5], [16], [17] and in phase I safety studies in healthy women [18] and men [19].

In order to predict the *in vivo* efficacy of a microbicide, there has been a focus on performing pharmacokinetic and pharmacodynamic studies to evaluate whether a candidate microbicide is bioavailable and present at levels sufficient to block HIV replication following vaginal application [20], [21], [22]. Efficacy studies of candidate microbicides against vaginal challenge of SHIV in nonhuman primate models indicate that microbicide concentrations that are required to prevent *in vivo* infection are orders of magnitude greater than the microbicide 50% effective concentrations (EC_50_) observed in cell culture [15], [23]. Furthermore, seminal plasma diminishes the ability of microbicide candidates to inhibit HIV and HSV [24], [25]. Thus, prior to performing microbicide efficacy studies in women it is critical to determine whether levels of microbicide that can be recovered after vaginal dosing are substantially in excess of the concentrations required to block viral replication in the absence and presence of seminal plasma. In addition, elucidating antiviral levels of microbicide that can be recovered as a function of time post-application is important for predicting whether the microbicide needs to be applied immediately prior to coitus or whether coitus can be delayed.

Here we describe the first clinical study of a topical microbicide that investigates *ex vivo* antiviral activity and local retention of SPL7013 Gel over a 24 h period following a single vaginal application of SPL7013 Gel in women. In addition, cervicovaginal fluid (CVF) samples were recovered by the participants using the SoftCup™ as an alternative to cervicovaginal lavage used in previous studies to evaluate *in vivo* microbicide levels [20], [21], [22]. The rationale for using the SoftCup was to enable self-sampling of the vaginal vault without introducing an unknown dilution normally associated with the cervicovaginal lavage method. The study was performed in healthy, sexually abstinent, HIV-uninfected women. Our data demonstrate that SPL7013 Gel was successfully recovered after vaginal dosing from all subjects and that high-levels of HIV-1 and HSV-2 inhibitory activity were observed in CVF samples in all participants up to 3 h post-dose, and in more than 50% of women at 24 h post-dose.

## Methods

### Study design

The protocol for this study and supporting CONSORT checklist are available as supporting information; see Checklist S1 and Protocol S1. The study was an open-label, single centre, randomised (for order of sampling with the treatment being the same in each case), 5-period, cross-over study, conducted between September 2008 and March 2009 at the clinical facilities of Nucleus Network [Alfred Medical Research and Education Precinct (AMREP) Centre for Clinical Studies], Melbourne, Australia. Study participants undertook five single applications of SPL7013 Gel, each dose separated by a minimum of 5 days. CVF samples were taken at 0 (within 2–10 min) (baseline), 1, 3, 12 or 24 h post-application in a randomised fashion using the INSTEAD^®^ SoftCup™. Additional CVF samples were collected at enrolment prior to dosing (pre-dose sampling period) for determination of intrinsic antiviral activity and assay validation. CVF samples were also collected at follow-up (approximately 1 week post final sample) for the determination of SPL7013 levels. Ethics approval was granted by the Alfred Human Research Ethics Committee (Drugs and Interventions Committee) on 31^st^ July 2008.

### Study participants

Female volunteers aged between 18–45 years were recruited by advertisement, and gave written informed consent to participate. Participants were eligible if they were not pregnant or breastfeeding, had a regular menstrual cycle, were in good general health as determined by medical history and physical/pelvic examination and Pap smear, did not have any sexually transmitted, urinary or vaginal infections, were not using any vaginal products, and were using a reliable form of contraception (*i.e.*, sterilisation, inter-uterine device or non-vaginal hormonal contraception).

### Study products

The study was open label. Study product was provided in pre-filled, single-use applicators containing 3.5 g of gel with 3% by weight (w/w) of SPL7013 (SPL7013 Gel). SPL7013 Gel is formulated using Carbopol 971P, a cross-linked acrylic acid listed in the USP as Carbomer 941 [4]. Participants self-administered study products at the clinical site, and all used applicators were retained and counted to check compliance.

### Study procedures

A screening visit was conducted up to 28 days prior to enrolment. During this visit a physical examination was performed, including a pelvic exam (with Pap smear if not performed in the previous year), vital signs and determination of body mass index (BMI). Samples were taken for HIV, HSV, hepatitis B virus, hepatitis C virus, *N. gonorrhoeae* and *C. trachomatis* screening, laboratory assessment (routine biochemistry, haematology and urinalysis), and pregnancy testing.

Women who fulfilled eligibility criteria and signed informed consent were enrolled in the trial. The SoftCup™ (Instead, Inc., La Jolla, CA, USA), a commercial menstrual collection device, was used to collect CVF samples, as previously described [26]. During the screening visit, each woman was trained in inserting and removing the SoftCup. Each participant was then provided with three CVF sampling kits (comprising a SoftCup, a 50 mL tube, an opaque bag marked with participant details and exact weight of the kit, and an instruction sheet), and discharged from the site. During the pre-dose sampling period (after the screening visit but before first dose of study product), participants provided three separate pre-dose CVF samples. Each sample was taken at least 24 h apart by inserting the SoftCup into the vagina, and then removing after 10–30 seconds. The used SoftCups containing CVF samples were then inserted in 50 mL sterile polypropylene tube and finally sealed in a labelled opaque envelope. Each pre-dose sample was obtained at the participant's home and refrigerated until being packed on ice and couriered to the study site within 24 h of collection. Once the pre-dose phase was complete, study participants attended six further visits to the clinical site. There was one visit each for Treatment Periods 1 to 5, and a follow-up visit at least one week after the final study treatment administration.

At Treatment Period 1 eligibility was confirmed, a urine pregnancy test performed, and each participant was randomised to one of five different sampling sequences. Each woman administered one applicator of SPL7013 Gel, and then obtained a CVF sample, as before, either immediately post-dose (within 2–10 min) (baseline); or at 1, 3, 12 or 24 h post-dose, depending upon the sampling sequence to which she was randomised. These procedures were repeated during Treatment Periods 2–5, such that a CVF sample was obtained for each woman at each of the 5 post-dose time-points.

Treatment Periods 1–5 were scheduled during the intra-menstrual period (at least 2 days prior to or 5 days after the expected onset of menses), and at least 5 days apart. Each post-dose CVF sample was obtained at the study site, and participants were permitted to leave the clinical unit between application and sample collection except when the baseline or 1 h samples were collected.

Participants were required to abstain from sexual and vigorous physical activity for 24 h prior to pre-dose sampling or product administration, and for the period between product application and post-dose CVF sampling. Participants were also required to refrain from administering vaginal preparations throughout the study period (except tampons, which were permitted for menstruation outside the product administration and sampling periods). The exception to this restriction was vaginal treatment of candidiasis or bacterial vaginosis products, which were permitted if prescribed by a medical practitioner. In such cases, study visits were to be suspended until 5 days after the cessation of treatment.

The final status of participants was determined at the follow-up visit, which included a physical exam (including vital signs and pelvic exam), laboratory assessment and a pregnancy test. The final CVF sample was also obtained at this visit for evaluation of SPL7013 levels. Throughout the study, data were collected on the participants' compliance with the study protocol, occurrence of adverse events (AEs), and administration of any concomitant medication.

### Storage and handling of the cervicovaginal samples

All CVF samples were stored at 4°C until processing, which was performed within 24 h of obtaining the study sample. The amount of recovered CVF was determining by calculating the difference between the combined weight of the 50 ml tube, the used SoftCup (with recovered CVF) and its original wrapper, and the combined weight of the unused SoftCup in its wrapper and the 50 ml tube. The viscosity of SPL7013 Gel made it difficult to quantitatively remove from the SoftCup. Therefore, to maintain consistency in the method used for recovery of the immediate pre-dose and post-dose samples, the CVF sample was washed from the SoftCup using aliquots of chilled 0.9% sterile phosphate buffered saline solution totalling 20 mL with shaking and low speed centrifugation to extract the maximum amount of sample from the cup. Ciprofloxacin and gentamicin were added to the clarified CVF samples to a final concentration of 5 µg/mL and 50 µg/mL respectively, for samples to be evaluated for HIV-1 and HSV-2 inhibitory activity. Samples without antibiotic were used for determination of the mass of SPL7013. All samples were stored at −80°C prior to analysis.

### Evaluation of SPL7013 mass and concentration

The determination of SPL7013 recovery in CVF samples was performed using a capillary zone electrophoresis method. The CVF samples were extracted from the SoftCups using a series of end-to-end rotation and centrifugation steps in the presence of isotonic saline solution. The SPL7013 and the internal standard, Orange G (Sigma-Aldrich, Castle-Hill, NSW, Australia), were monitored at 240 nm on a Beckman P/ACE MDQ capillary electropherograph (CE) equipped with a diode array detector. The concentration of SPL7013 in the unknown samples was determined using a calibration curve derived from samples of known SPL7013 concentrations. Total mass of SPL7013 present in the original sample was determined by correcting for the dilution during processing of the sample. The lower limit of quantitation for the SPL7013 recovery from the SoftCup and subsequent analysis was 2.25 mg.

### Evaluation of HIV-1 inhibitory activity

The HIV-1 inhibitory activity of pre- and post-dose CVF samples was determined against HIV_Ba-L_, a laboratory clade B strain that utilises the CCR5 chemokine coreceptor for viral entry [27]. Assays were performed in the TZM-b1 indicator cell line using luciferase as the readout for HIV replication [5]. Tissue culture plates (96-well, Nunc) were seeded with 20,000 cells per well the day prior to assay. CVF samples were then diluted 1∶2, 1∶4 and 1∶20 in Opti-MEM (Gibco) containing 0.05 µg/mL amphotericin B (which does not inhibit HIV-1 replication at this concentration, data not shown), 2.5 µg/mL ciprofloxacin, 50 µg/mL gentamicin and 20 µg/mL DEAE-dextran, added to triplicate wells and the plates incubated for 30 min at 37°C in a 5% CO_2_ humidified incubator. HIV-1_Ba-L_ virus stock, propagated in PBMCs from HIV seronegative donors, was then added at 500 infectious units (IU)/well and the plates incubated for a further 2 days. HIV-1 replication was assessed by determination of luciferase activity in cell lysates using the Steady-Glo Luciferase Assay System (Promega). Virus only and cell controls were included to determine maximum levels of HIV-1 replication and background levels of luciferase activity, respectively. The percentage inhibition of HIV-1 replication in pre-dose and post-dose samples was calculated by dividing the average luciferase activity obtained from triplicate wells (after subtracting background luciferase activity from uninfected cells) by the luciferase activity obtained from untreated HIV-1_Ba-L_ infected cells. Unformulated SPL7013 was used as a positive control, and assays were considered valid if the EC_50_ fell within two standard deviations of the mean of previously performed assays (5.0±5.2 µg/mL, n = 8). The SPL7013 EC_50_ value was calculated from nonlinear regression analysis of the data as described previously [5]. Cytotoxicity of CVF samples was determined at the same time in duplicate plates to which CVF dilutions but no virus were added. Cytotoxicity was assessed using the MTS reagent (CellTiter 96 Aqueous One, Promega) as previously described [5]. CVF samples that resulted in cell viability of less than 80% compared to the cell control were considered cytotoxic and were not considered for effects on HIV-1 replication. Three independent valid assays were performed for each CVF sample.

### Evaluation of HSV-2 inhibitory activity

The HSV-2 inhibitory activity of CVF samples was determined in human embryonic lung (HEL) fibroblasts using cell viability as the readout for viral replication. The HSV-2 isolate (#250733) [5] that was used in the assay is a highly cytopathic clinical isolate obtained from an Australian patient and isolated and typed by the Victorian Infectious Diseases Reference Laboratory (VIDRL, Melbourne, Australia). Tissue culture plates (96-well) were seeded with 6,000 cells per well a day prior to assay and then triplicate wells were incubated with CVF samples, diluted 1∶2, 1∶5 and 1∶50 in DMEM (Gibco) containing 2 mM glutamine, 25 mM HEPES, 2.5 µg/mL amphotericin B, 10 µg/mL ciprofloxacin, 50 µg/mL gentamicin and 2% foetal calf serum, for 30 min at 37°C in a 5% CO_2_ humidified incubator, followed by addition of HSV-2 (560 TCID_50_/well). The plates were incubated for a further 5 days before HSV-2 replication was assessed by cell viability using the MTS reagent. Virus and cell only controls were included and cytotoxicity of the CVF dilutions determined as above. CVF samples that resulted in cell viability of less than 70% compared to the cell control were considered cytotoxic and were not considered for effects on HSV-2 replication. The definition of cytotoxicity for HEL cells differed to that used for TZM-bl cells due to the sensitivity of HEL cells to >100 µg/ml of SPL7013 (data not shown). The percentage inhibition of samples was calculated by subtracting the average optical density (OD) of samples relative to cell only controls (after subtracting virus only controls) from 100. Unformulated SPL7013 was used as a positive control, and assays were considered valid if the EC_50_ fell within the range 1.0±1.3 µg/ml (mean ±2SD, n = 5). Again, three independent valid assays were performed for each CVF sample.

### Antiviral activity in the presence of seminal plasma

CVF samples from participants 1, 3 and 5, which represented examples of short, long and intermediate retention times of complete viral inhibition, respectively, were further analysed in the presence of pooled seminal plasma from four HIV seronegative donors (Lee BioSolutions Inc, St. Louis, Missouri). For the evaluation of HIV-1 inhibitory activity, samples were diluted 1∶2 and incubated with TZM-bl cells as above. HIV-1_Ba-L_ was then added along with seminal plasma to give a final seminal plasma concentration of 12.5%, a level shown to be non-toxic to TZM-bl cells (data not shown). To achieve a similar level of luciferase activity after 2 days incubation to that seen without seminal plasma, a 4-fold higher inoculum of HIV-1_Ba-L_ was required. Similar controls to those described above for evaluation of anti-HIV-1 activity were again included and three independent assays performed.

For the evaluation of HSV-2 inhibitory activity, CVF samples were diluted to 1∶50 and added to HEL cells as described above. HSV-2 was then added in the presence of a final concentration of 10% seminal plasma and the cells incubated for 5 days as described above. 10% seminal plasma was shown to be non-toxic to HEL cells over this period. For HSV-2 assays, a 2-fold higher inoculum was required compared to assays performed in the absence of seminal plasma to achieve the same level of baseline infection.

### Contribution of SPL7013 to the antiviral activity of SPL7013 Gel

To determine the contribution of SPL7013 to the antiviral activity of SPL7013 Gel present in the CVF samples of participants, 2 g of SPL7013 Gel or Placebo Gel (being the same formulation but absent of SPL7013) and 0.5 g of pooled CVF samples from four HIV seronegative donors (Lee BioSolutions) were thoroughly mixed with 20 mL saline to mimic the maximum amount of gel recovered in post-dose samples, and the average amount of CVF collected in pre-dose samples. Following centrifugation, as for the recovery of CVF samples, the gel/CVF mixtures were compared for their anti-HIV-1 activity at 1∶2, 1∶4, 1∶20, 1∶100 and 1∶200 dilutions in TZM-bl cells, and for anti-HSV-2 activity at 1∶2, 1∶5, 1∶50, 1∶100 and 1∶200 dilutions in HEL cells, as described above. Cytotoxicity of the dilutions in the two cell lines was also assessed using the MTS reagent. Three independent assays were performed with each virus.

### Assessment of safety

Safety was assessed from reports of adverse events (AEs), vital signs and routine laboratory assessment. An AE was described as any untoward medical occurrence in a subject administered an investigational product, and which does not necessarily have a causal relationship to the study drug. All AEs reported during the study were graded according to the Division of AIDS Genital Grading Table for Use in Microbicide Studies and the Division of AIDS Table for Grading the Severity of Adult and Paediatric Adverse Events, including Addendum 1 Female Genital Grading Table for Use in Microbicide Studies [28] and assigned a potential causality to study product.

### Statistical methods

The sample size was considered typical of a study assessing pharmacokinetic parameters as the primary endpoints, and was also similar to sample sizes employed in the two similar studies assessing antiviral activity of the microbicide PRO 2000 [20], [21]. Comparison of parameters for the 1, 3, 12 and 24 h CVF samples with those for the baseline sample, were performed initially using a Friedman test to assess overall differences between timepoints. Pairwise comparisons were then assessed using the Wilcoxon signed-rank test for repeated measures data with Bonferroni adjustment, where p≤0.003 was statistically significant (STATA version 11, StataCorp, College Station, TX, US). The impact of seminal plasma and relative effects of placebo compared to SPL7013 Gel on antiviral activity was performed using the Wilcoxon signed-rank test (one-sided) (GraphPad PRISM version 4, GraphPad Software, San Diego, CA, USA), where p<0.05 was considered statistically significant. In the case of non-significant associations/differences, with the limited sample size of the study, there is insufficient evidence to be able to demonstrate a difference, if indeed such a difference does truly exist.

## Results

### Enrolment and adverse events

Twelve eligible women were enrolled in the study. Ten completed CVF sampling for all 5 time-points. An additional woman completed four time-points, including the baseline sample, and therefore was included in the Per Protocol analysis. One of the 12 participants was lost to follow up due to reasons unrelated to AEs or study procedures. Participants were aged between 19–37 years old (mean 25 years old), with a BMI ranging from 18.2 to 30.3 (mean 23.5). All women were using hormonal contraception at baseline, and continued throughout the study.

There were no clinically significant changes in systemic laboratory parameters during the study. No grade 3 or 4 AEs, serious AEs or deaths were reported during the study. Eight non-genitourinary AEs (mainly grade 1) were experienced by six subjects (50%), all of which were considered not related to study product. Seven genitourinary AEs were experienced by four subjects (33.3%) who received at least one dose of 3% SPL7013 Gel. Two of the AEs reported were considered likely related to SPL7013 Gel administration and were perineal irritation (Grade 1) and bacterial vaginosis (Grade 2). The only reported AEs considered to be possibly related to 3% SPL7013 Gel administration were three cases of clinical symptoms of vaginal candidiasis in three subjects (Grade 1). All other AEs were deemed unlikely to be related to SPL7013 Gel and were Grade 1 in severity. No trends were observed for the time of onset of AE, or randomisation sequence for CVF sampling time.

### Recovery of CVF sample using the SoftCup self-sampling method

**Figure 1:**
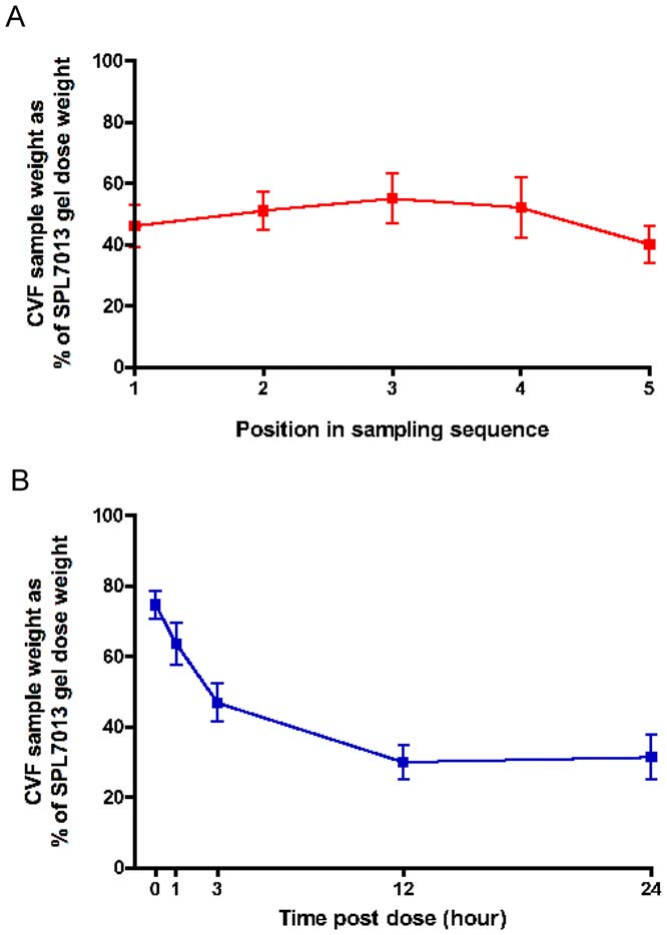
Recovery of CVF samples using the SoftCup. Weight of post-dose CVF samples as a percentage of the actual weight of SPL7013 Gel administered vaginally compared to the position in the sampling sequence (A) and the time post-dose (B). Data represent the average from the eleven study subjects. Error bars denote standard error of the mean.

The average weight ± standard deviation of CVF sample recovered pre-dose from 11 participants was 0.44±0.27 g (range 0.14–1.06 g), which is similar to a previous study [26]. The mean % (standard error) recovery of post-dose CVF sample (including gel) compared to the weight of SPL7013 Gel application (3.5 g) for first, second, third, fourth and fifth samples (*i.e.*, the randomized sequence in which each sample was taken irrespective of the time-point) for all participants was 46% (6.7), 51% (6.3), 55% (8.3), 52% (9.8) and 40% (6.2), respectively (Figure 1A). There were no significant differences in recovery between the post-dose samples (p>0.20 in each case, Wilcoxon signed-rank test), indicating consistency of sampling.

We also evaluated the mean % weight of the recovered post-dose CVF samples compared to the weight of the original sample of SPL7013 Gel with respect to time post dose (Figure 1B). A time-dependent decrease in the weight of recovered CVF was observed with a mean 75% of the original sample recovered at baseline, which plateaued at 12 h (30%) and 24 h (31%) post-dose. These data demonstrate consistent collection of CVF samples using the SoftCup and a time dependent decrease in the weight of recovered sample.

### Recovery of SPL7013 using the SoftCup self-sampling method

**Figure 2:**
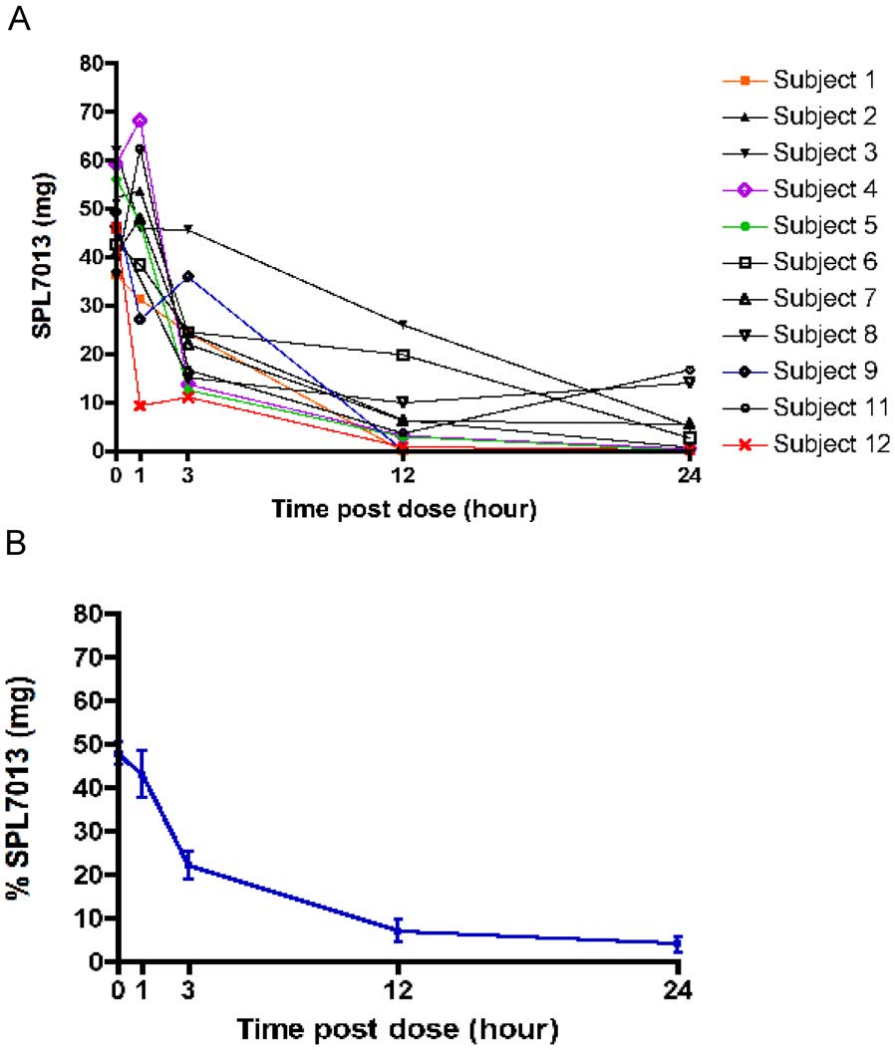
Recovery of SPL7013 using the SoftCup. Mass of SPL7013 (mg) recovered in CVF samples from each subject taken at baseline (within 2–10 minutes post-dose) (0), 1, 3, 12 and 24 h post-dose (A). Average recovery of SPL7013 as a percentage dose of SPL7013 administered (105 mg) (B). Error bars denote standard error of the mean.

We determined the mass of SPL7013 that was recovered compared to the mass of SPL7013 dosed (*i.e.*, 105 mg) for each participant at each time point (Figure 2A). At baseline, a 48 mg of the original dose (mean) was recovered representing 46% of the original mass of SPL7013 applied to the vagina (Figure 2B). The mean % recovery compared to the original mass of SPL7013 at times 1, 3, 12 and 24 h post dose was 42, 21, 6.8 and 3.9%, respectively (Figure 2B). This represents 90, 46, 15 and 9% recovery compared to the baseline post-dose CVF sample, respectively. These data demonstrate that the mass of recovered SPL7013 decreases with time and that a large proportion of the original SPL7013 (>20%) dose can be recovered at 1 and 3 h following SPL7013 Gel administration.

### HIV-1 inhibitory activity of pre- and post-dose CVF samples

**Figure 3:**
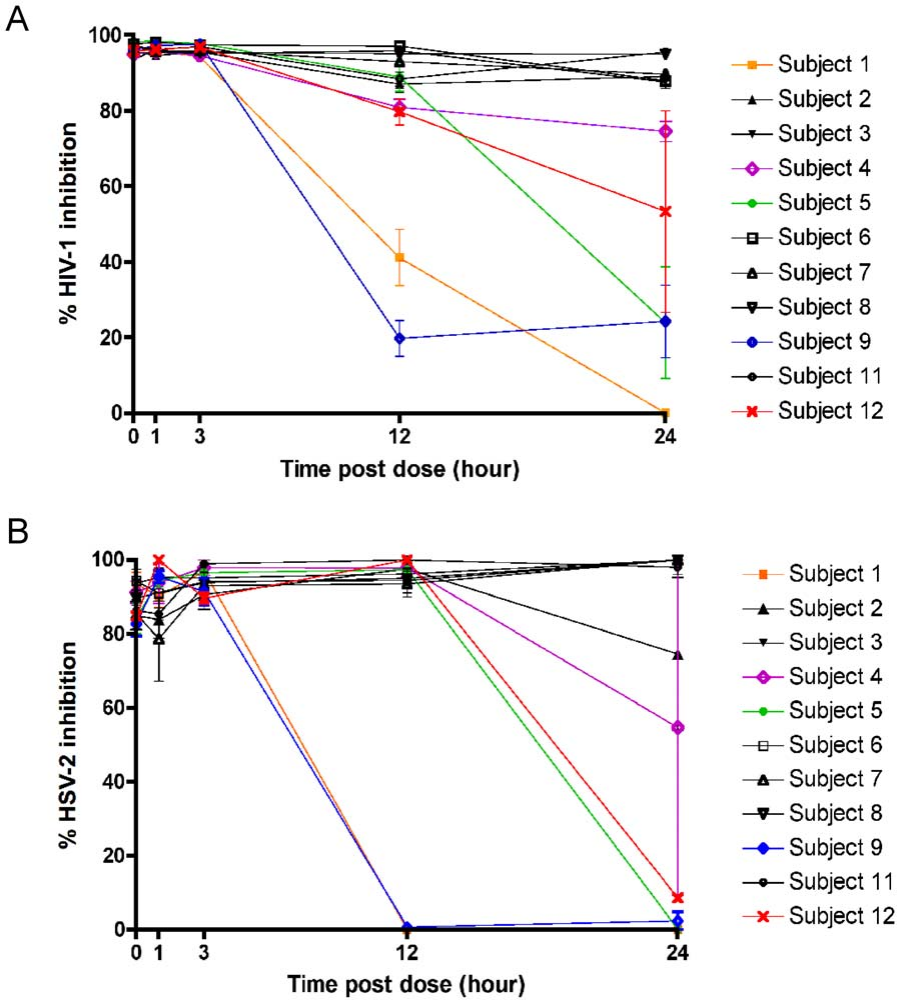
HIV-1 and HSV-2 inhibitory activity of post-dose CVF samples. The percent inhibition of HIV-1 replication mediated by post-dose CVF samples from each subject compared to HIV-1 infected cells incubated in the absence of CVF sample. All samples recovered in 20 mL of saline were diluted an additional 1∶2 (total dilution of samples ∼ 1∶40) (A). The percent inhibition of HSV-2 replication mediated by post-dose CVF samples from each subject compared to HSV-2 infected cells incubated in the absence of CVF sample. All samples recovered in 20 mL of saline were diluted an additional 1∶50 (total dilution of samples ∼ 1∶1000) (B). Data are from three independent assays. Error bars denote standard error of the mean.

The ability of pre- and post-dose samples to inhibit HIV-1 replication was determined in the TZM-bl indicator cell line. CVF samples recovered in 20 mL of saline were subjected to an additional 1∶2 dilution in medium, representing a total dilution of ∼1∶40 of the neat CVF sample, which was not cytotoxic to TZM-bl cells used to evaluate HIV-1 inhibitory activity. Since CVF samples obtained at the final non-dosing study visit were found to have undetectable levels of SPL7013, they were not evaluated for antiviral activity. The median (interquartile range) inhibition of HIV-1 by pre-dose CVF samples was 1.9% (0, 5.6). In contrast, almost complete HIV-1 inhibition was observed with baseline CVF samples (Figure 3A), where a median (interquartile range) inhibition of 96% (95, 97) was achieved. The 1, 3, 12 and 24 h CVF samples provided a median inhibition of 96% (95, 99) (p = 0.77), 96% (94, 98) (p = 0.91), 88% (20, 97) (p = 0.003) and 88% (0, 96) (p = 0.001), respectively. Inhibition of HIV replication at 1 and 3 h post-dose was not significantly different (defined as p≤0.003, Bonferroni adjusted p-value significance level) compared to the baseline CVF sample. There was a significant drop in median inhibition observed at 12 and 24 h post-dose, which was due to reductions in the ability of recovered CVF samples from five women to inhibit HIV-1 (Figure 3A).

### HSV-2 inhibitory activity of pre- and post-dose CVF samples

The pattern of HSV-2 inhibitory activity was very similar to that for HIV-1 (Figure 3B). Anti-HSV-2 activity was low at pre-dose, but almost complete at baseline with median inhibitions of 0.6% (0, 1.5) and 86% (85, 94), respectively. Median HSV-2 inhibition for the 1, 3, 12 and 24 h samples was 92% (79, 100) (p = 0.237), 94% (90, 99) (p = 0.005), 96% (0,100) (p = 0.027) and 75% (0, 100) (p = 0.756), respectively. Similar to HIV-1, there was no significant difference in anti-HSV-2 activity (defined as p≤0.003, Bonferroni adjusted p-value significance level) at 1 and 3 h post-dose compared when data was analysed using the Wilcoxon signed-rank test with Bonferroni adjustment. In contrast to HIV-1, the difference between median inhibition at baseline and that seen at either 12 or 24 h did not reach, but tended towards, statistical significance for HSV-2. In contrast to the HIV-1 inhibition assays, CVF samples recovered in 20 ml of saline were diluted an additional 1 in 50 for evaluation of HSV-2 inhibitory activity since this was the lowest dilution that was noncytotoxic to the HEL cells used in the assay.

### Impact of seminal plasma on HIV-1 and HSV-2 inhibitory activity of post-dose CVF samples

**Figure 4:**
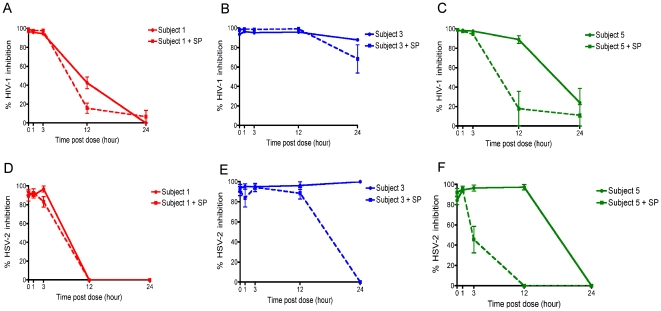
Impact of seminal plasma on the HIV-1 and HSV-2 inhibitory activity of post-dose CVF samples. Inhibition of HIV-1 replication by post-dose CVF samples from subjects 1 (A), 3 (B) and 5 (C) performed in the absence and presence of 12.5% seminal plasma (SP). Inhibition of HSV-2 replication by post-dose CVF samples from subjects 1 (D), 3 (E) and 5 (F) performed in the absence and presence of 10% seminal plasma. Data are from three independent assays. Error bars denote standard error of the mean.

Seminal plasma can mediate adverse effects on the antiviral activity of microbicides [24], [25]. We selected CVF samples from participants displaying three different patterns of HIV-1 and HSV-2 inhibition representing short (subject 1), intermediate (subject 5) and long (subject 3) retention times of complete inhibition (Figure 4). These samples were tested to determine whether the addition of seminal plasma diminished inhibition of viral replication compared to samples that we had tested in the absence of seminal plasma.

We did not observe a decrease in the ability of CVF samples from the three subjects to block HIV-1 replication compared to baseline at 1 and 3 h post-dose in the presence of seminal plasma compared to no seminal plasma (Figure 4A, B and C). One exception was the 3 h post-dose sample for subject 5, where the decrease in HIV-1 inhibition observed in the presence of seminal plasma tended towards significance (p = 0.054, n = 3, Wilcoxon signed-rank test) and was minimal in the context of this type of biological assay (average decrease of 4.5%) (Figure 4C). In addition, seminal plasma did not decrease the HIV-1 inhibitory activity of CVF samples from subject 3 obtained at 12 and 24 h post-dose (Figure 4B). In contrast, seminal plasma conferred a significant decrease in HIV-1 inhibition, which tended towards significance with CVF samples from subjects 1 and 5 taken at 12 h post-dose (p = 0.062, n = 3) (Figure 4A and C).

A similar pattern was also observed for inhibition of HSV-2 for subjects 1 and 3, where there was no significant decrease of HSV-2 inhibitory activity in the presence of seminal plasma compared to no seminal plasma with CVF samples taken at baseline, 1 and 3 h post-dose (Figure 4D and E). Subject 5 was the exception, where there was a drop in HSV-2 inhibition tending towards significance for the 3 h sample (p = 0.062, n = 3) (Figure 4F). These data demonstrate that for the majority of patients, seminal plasma had little effect on the ability of the CVF samples to inhibit HIV-1 and HSV-2 replication at up to 3 h post-dose.

### Relationship between recovered SPL7013 mass or concentration and HIV-1 and HSV-2 inhibitory activity in CVF samples

**Figure 5:**
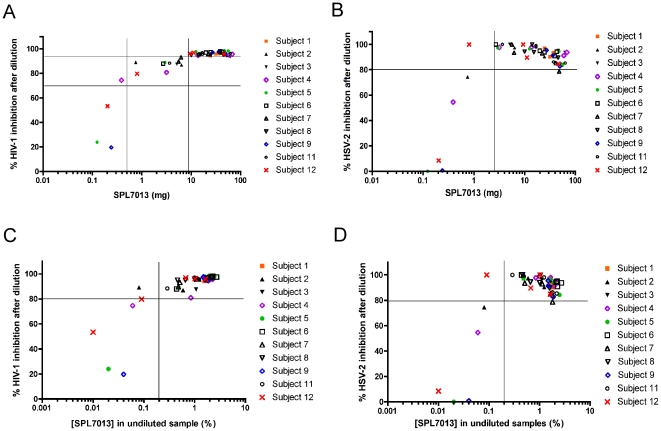
Relationship between recovered mass and concentration of SPL7013, and HIV-1 and HSV-2 inhibitory activity of post-dose CVF samples. The inhibition of viral replication in post-dose CVF sample from all participants as a function of either the mass of recovered SPL7013 for HIV-1 (A) and HSV-2 (B) or the concentration of SPL7013 in the original undiluted samples for HIV-1 (C) and HSV-2 (D).

We plotted the total mass of recovered SPL7013 against HIV-1 (Figure 5A) and HSV-2 inhibition (Figure 5B) mediated by post-dose CVF samples. The inhibition of HIV-1 (Figure 5C) and HSV-2 (Figure 5D) as a function of SPL7013 concentration was also analysed. High levels of anti-HIV-1 activity (*i.e.*, >94% inhibition) were observed when >9 mg of SPL7013 was present in CVF samples and >70% inhibition was achieved with as little as 0.5 mg of SPL7013. These levels represent 8.6% and 0.5% of the total mass of SPL7013 applied to the vagina, respectively (Figure 5A). High levels of anti-HSV-2 activity (*i.e.*, ≥80% inhibition) was observed with >2.5 mg of SPL7013, representing 2.4% of the original dose (Figure 5B). A lower cut off for maximal inhibition of HSV-2 compared to HIV-1 replication was necessary due to cytotoxicity to HEL cells mediated by high concentrations of SPL7013 (>35 mg) that was also observed in our pilot studies. Greater than 80% HIV-1 (Figure 5C) and HSV-2 (Figure 5D) inhibition was observed if at least 0.2% of the dosed SPL7013 was present in the original CVF sample. This represents ∼50 ng/ml of SPL7013 when taking into consideration the ∼1∶40 dilution of the samples for HIV-1, and ∼2 ng/ml when taking into account the ∼1∶1000 dilution required for testing CVF samples in the HSV-2 assays. These data show that SPL7013, at levels representing a fraction of the original dose, demonstrates high levels of HIV-1 and HSV-2 inhibitory activity in *ex vivo* assays despite extensive dilution of the CVF samples by ∼1∶40 and ∼1∶1000, respectively.

### Contribution of SPL7013 to the antiviral activity of SPL7013 Gel

**Figure 6:**
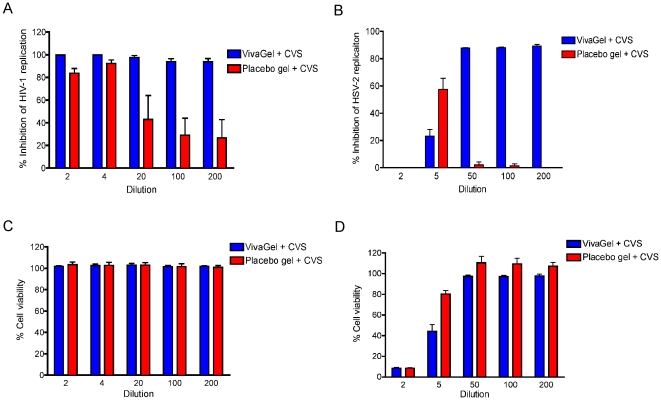
Contribution of SPL7013 to antiviral activity in SPL7013 Gel. Inhibition of HIV-1 (A) and HSV-2 (B) replication of 2 g of SPL7013 Gel or Placebo Gel combined with 0.5 g of pooled human cervicovaginal secretions and diluted in 20 mL of saline. Cytotoxicity of the samples in TZM-bl cells (C) and HEL cells (D). Dilution denotes fold dilutions of CVF sample in medium that were initially recovered in saline. Data are from three independent assays. Error bars denote standard error of the mean.

The Placebo Gel is identical to SPL7013 Gel except for the absence of SPL7013. Thus, the Placebo gel comprises a Carbopol vehicle at pH 4.5, so the acidity of this gel could impart virucidal activity [29], [30], [31]. Therefore, to determine the contribution of SPL7013 in the SPL7013 Gel formulation to the antiviral activity of CVF samples obtained in the study, *in vitro* experiments with Placebo Gel were performed to mimic the assays performed with CVF samples from the trial participants. Either 2 g of either SPL7013 Gel or Placebo Gel were combined with 0.5 g of pooled CVF samples. These samples where then processed as described for the participant CVF samples, except that we investigated additional sample dilutions (Figure 6A and B). In parallel, assays were performed to determine doses that were not toxic to the cells used in the antiviral assays to identify dilutions that could be evaluated for antiviral activity (Figure 6C and D).

The data reported here show similar levels of HIV-1 inhibition by SPL7013 Gel and Placebo Gel diluted at 1∶2 and 1∶4 (Figure 6A). In contrast, ≥90% HIV-1 inhibition by SPL7013 Gel was observed at 1∶20, 1∶100 and 1∶200 dilution while inhibition was lower in the presence of Placebo Gel, which tended towards significance (p = 0.05). No cytotoxicity was observed for either gel formulation at any of the dilutions tested (Figure 6C). The HIV-1 inhibitory activity of post-dose CVF samples that were diluted 1∶20 compared to the 1∶2 dilution (Figure 3A) demonstrated similar high levels of HIV inhibition (≥85%) for all participants up to 3 h post-dose (data not shown). These data indicate that the HIV-1 inhibitory activity of CVF samples can be observed at dilutions where SPL7013 Gel (and thus SPL7013), but not Placebo Gel is inhibitory (Figure 6A).

Significant cytotoxicity is observed in cells used in the HSV-2 inhibitory assays at 1∶2 and 1∶5 dilutions for both SPL7013 Gel and Placebo Gel (compromising the ability to assess HSV-2 inhibition at these dilutions), but not at the 1∶50 dilution or greater (Figure 6D). Cytotoxicity was absent at and above 1∶50 dilution and these dilutions could thus be evaluated for anti-HSV-2 activity. SPL7013 Gel demonstrated high levels of HSV-2 inhibition compared to Placebo Gel, which lacked inhibitory activity at 1∶50, 1∶100 and 1∶200 dilutions (Figure 6B). Since post-dose CVF samples from all participants were tested at a 1∶50 dilution in the anti-HSV-2 assays (Figure 3B), the inhibition observed in our cell culture assays is almost certainly due to SPL7013 and not the vehicle (Placebo Gel). Taken together, these data indicate that both SPL7013 and Placebo Gel are likely to contribute to HIV-1 inhibitory activity, however SPL7013-containing gel retains HIV-1 and HSV-2 inhibitory activity upon significant dilution.

## Discussion

This study demonstrates for the first time the retention of HIV-1 and HSV-2 inhibitory levels of SPL7013 Gel in the female genital tract over a 24 h period. The strength of this study compared to previous studies of microbicide vaginal pharmacokinetics and pharmacodynamics [20], [21] is that we followed the time course of retention of antiviral activity present in recovered CVF samples after a single dose of microbicide, in addition to determining the concentrations of SPL7013 present in recovered CVF samples with time. Furthermore, the SoftCup was effectively used for the recovery of CVF samples instead of cervicovaginal lavage, thus allowing direct calculation of the degree of sample dilution. Our data show that the minimum retention time, consistent with high level HIV-1 and HSV-2 inhibitory activity in the post-dose CVF samples in all participants, was 3 h after a single dose of SPL7013 Gel. Remarkably, the CVF samples from the majority of participants (6/11) also demonstrated high levels (≥80% inhibition) of antiviral activity at 24 h post-dose. Quantitation of the mass of SPL7013 recovered from CVF samples showed that >9 mg and >2.5 mg of SPL7013 was associated with high levels of HIV-1 and HSV-2 inhibition, respectively, representing a small fraction of the original 105 mg dose of SPL7013 in SPL7013 Gel. Notably, antiviral activity was retained in the presence of seminal plasma for the majority of the participants up to 3 h post-dose indicating that dosing of SPL7013 Gel up to 3 h prior to coitus could be incorporated in clinical trials evaluating microbicide efficacy.

Consistent with previous phase I safety studies, SPL7013 Gel demonstrated no serious AEs, nor Grade 3 or 4 AEs, and the genitourinary AEs were mainly mild in severity [18], [19]. No trends were observed for the time of onset of AE or randomisation sequence for CVF sampling time and there were no withdrawals due to AEs.

Remarkably, prolonged retention of HIV-1 and HSV-2 inhibitory activity was observed in the CVF samples from the majority of participants at 12 (8/11) and 24 h (6/11) post-dose. SPL7013 is formulated in a Carbopol vehicle that has mucoadhesive properties, which may extend its retention in the female genital tract. However, it is not clear why some women demonstrated prolonged retention of SPL7013 compared to others. The women, while ambulatory, were instructed to abstain from sex and not undertake physical exercise, such as running, that could promote clearance of the gel from the vagina. Natural variations in anatomical shape of the vagina may have contributed to the difference in retention. It is also possible that women experiencing lower SPL7013 retention times may have experienced vaginal child-birth, thus weakening the pelvic floor. However, such data was not collected from the participants and therefore it would be interesting to examine this possibility in future studies.

A limitation of this study is that, in order to determine HIV-1 and HSV-2 inhibitory levels in recovered pre- and post-dose CVF samples, it was necessary to dilute the samples. This was because the assays were cell culture based and our pilot studies demonstrated that cervicovaginal secretions, the gel vehicle and moderately high concentrations of SPL7013 (in the case of HEL cells) reduced cell viability and therefore would interfere with the evaluation of antiviral levels in the CVF samples. Accordingly, we performed antiviral and cytotoxicity assays in parallel and three independent assays to obtain robust data for analysis. The total dilution of the 1∶2 and 1∶50 CVF samples tested in the HIV-1 and HSV-2 inhibition assays was approximately 1∶40 and 1∶1000 respectively, after taking into consideration dilution of the sample with 20 mL of sterile saline that was essential for quantitative removal of sample from the SoftCup. This compares to final (estimated) dilutions of 1∶20 and 1∶40 in PRO 2000 samples recovered after vaginal application by cervicovaginal lavage using 10 mL of saline [20]. Given the considerable dilution of CVF samples, virus inhibition observed for pre- and post-dose samples are likely to be an underestimate of the actual activity and quantity of the product in the genital tract. CVF sample dilution could also explain why we did not observe intrinsic HIV-1 [32] and HSV-2 [33] inhibitory activity in pre-dose CVF samples from all participants similar to that observed in a previous study with PRO 2000 [20]. Extensive dilution of our CVF samples is also likely to underestimate the length of duration of high levels of antiviral activity.

PRO 2000 is a linear macromolecular polyanion microbicide, which has undergone similar studies to determine the retention of antiviral levels of microbicide after intravaginal dosing in women, except that samples were recovered by cervicovaginal lavage with 10 mL of saline and only single time points were evaluated [20], [21]. For the 0.5% PRO 2000 dose, 6.6% recovery of PRO 2000 was achieved for the 2 h post-dose samples [21] and 5.8 – 17% recovery for 0.5% PRO 2000 at 1 h post-dose [20] using the same calculation as described by Lacey and colleagues [21]. Using the SoftCup we achieved an average 41% recovery (50 mg) of the original 105 mg dose of SPL7013 in the 1 h post-dose CVF samples, which is substantially greater than the highest recovery reported for 0.5% PRO 2000. In addition, the baseline post-dose recovery of SPL7013 was 46%. The SoftCup method's recovery of a robust fraction of the recently applied SPL7013 increases confidence in its effective recovery of the intraluminal drug. The SoftCup has a soft elastomeric rubber rim surrounding a collection dome, which when inserted and removed from the vagina has a spatula-like action that may more efficiently collect viscous fluids from the vaginal lumen than does cervicovaginal lavage. However, complete recovery of SPL7013 Gel was not achieved even for baseline post-dose CVF samples. SPL7013 loss is unlikely to be due to systemic absorption [4]. Thus, SPL7013 Gel may have been lost due to leakage from the vagina, inaccessibility to the SoftCup or remained non-specifically bound to the epithelial cells lining the female genital tract. Our data also show that use of the SoftCup leads to reproducible sample recovery independent of the sampling sequence, allows direct calculation of the extent of sample dilution, and can be self administered by the participant following minimal instruction, thus representing a promising alternative to cervicovaginal lavage.

Levels of PRO 2000 that were sufficient to block HIV-1 infection in cell culture assays were recovered from retention studies with 0.5% PRO 2000 [20], [21]. However, this formulation failed to demonstrate significant protection in preventing HIV acquisition in phase IIB and III clinical trials [34], [35]. The dendrimer, SPL7013, has a chemically defined structure and a distinct surface group compared to polymeric linear polyanion-based microbicides such as PRO 2000. In the 3.5g dose of 3%w/w SPL7013 Gel there is an approximate 20,000-fold excess of SPL7013 delivered to the female genital tract compared with the *in vitro* EC_50_ for HIV clinical isolates and an approximate 45,000-fold excess of SPL7013 compared with the *in vitro* EC_50_ for a HSV-2 clinical isolate [5]. Compared to 2 g of 0.5% PRO 2000 [21], SPL7013 Gel delivers a 10.5-fold higher mass of active drug at levels that are tolerated *in vivo* [18]. Our retention study shows that in baseline, 1 and 3 h post-dose CVF samples we were able to recover an average 48, 43 and 22 mg of SPL7013, respectively. This represents SPL7013 levels (even when diluted in semen and CV secretions) that are at least 10^3^-fold greater than the cell culture EC_50_ for inhibition of both HIV-1 and HSV-2. In previous studies, a 1∶30 dilution of SPL7013 Gel retained anti-HIV activity in the presence of seminal plasma retained anti-HIV activity in cell culture assays [9]. Consistent with this observation, the HIV-1 and HSV-2 inhibitory activities of recovered CVF samples in this study remained high for the majority of participants up to 3 h post dose even when assays were performed in the presence of seminal plasma. However, other factors that could affect the efficacy of a gel based macromolecular microbicide, apart from dilution or inhibition by genital secretions, includes its re-distribution within the vagina, which may also be affected by stirring during the act of coitus [22], which was not assessed in this study. As significant inhibition of HIV-1 and HSV-2 is achieved with a small percentage of the original dose of SPL7013, it can be theorised that the effects of coitus would not significantly impact the activity of the product. However this could only be established with further clinical investigation. Nevertheless, in the proof of concept study demonstrating that a topical microbicide gel containing 1% tenofovir protected women against HIV acquisition with 39% efficacy compared to placebo gel, more HIV negative women had detectable CVF concentrations of tenofovir, and tenofovir concentrations in CVF were associated with reduced risk of HIV and HSV-2 infection [36].

In conclusion, we have demonstrated retention of high-level HIV-1 and HSV-2 inhibitory activity of a dendrimer microbicide following vaginal administration that is long-lived in the majority of participants. While efficacy trials will be required to determine whether SPL7013 Gel is able to protect women against HIV and HSV acquisition, the concentration of drug in CVF samples is a potentially important means to determine target drug concentrations for providing protection and is a potential surrogate marker for protection.

## Supporting Information

Protocol S1 Study Protocol.
(DOC)

Checklist S1 CONSORT checklist.
(DOC)
