# Supplementary material for: SPL7013 Gel (VivaGel®) Retains Potent HIV-1 and HSV-2 Inhibitory Activity following Vaginal Administration in Humans
Source: PLoS One. 2011 Sep 15;6(9):e24095. doi: 10.1371/journal.pone.0024095 (PMC3174146; doi:10.1371/journal.pone.0024095)
Supplement: Protocol S1 — Study Protocol. (DOC) [file pone.0024095.s001.doc]

#
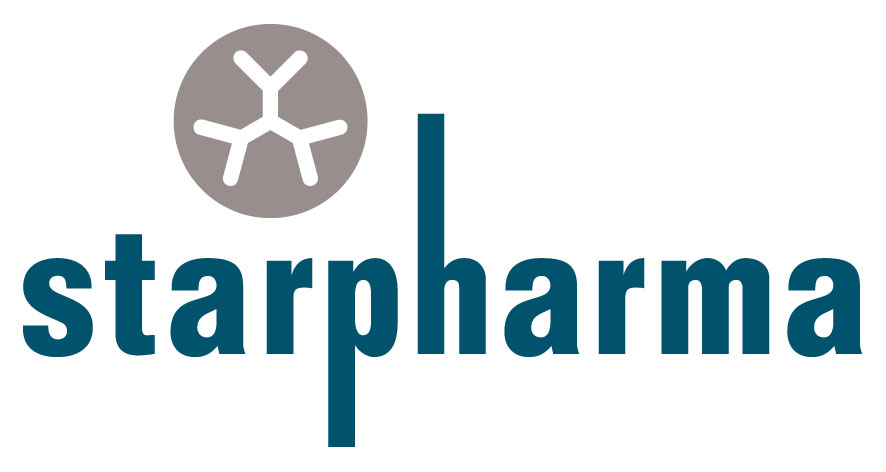


# Assessment of local retention and duration of activity of SPL7013 following vaginal application of 3% SPL7013 Gel (VivaGel®) in Healthy Volunteers

| **Protocol Number:** |  | SPL7013-003 |
| --- | --- | --- |
|  |  |  |
| **Investigational Product:** |  | SPL7013 Gel |
|  |  |  |
| **IND Number:** |  | 62,482 |
|  |  |  |
| **Sponsor:** |  | Starpharma Pty Ltd  Baker Building  75 Commercial Road  Melbourne Victoria 3004  Australia |
|  |  |  |
| **Sponsor Clinical Representative:** |  | Clare Price  Clinical Development Manager |
|  |  |  |
| **Financial Sponsor:** |  | National Institutes of Health (NIH)  National Institute of Allergy and Infectious Diseases (NIAID)  6700-B Rockledge Dr, MSC 7612  Bethesda, Maryland 209892-7612, USA  Contract No. HHSN266200500042C  ADB Contract No. N01-AI-50042 |
|  |  |  |
| **Principal Investigator:** |  | Dr Peter Hodsman  Nucleus Network Ltd  5^th^ Floor, Burnet Tower  89 Commercial Road  Melbourne Victoria 3004  Australia |

# CONFIDENTIAL

This protocol includes information and data that contain trade secrets and privileged or confidential information which is the property of Starpharma Pty Ltd (“Starpharma”). This information must not be made public without written permission from Starpharma. These restrictions on disclosure will apply equally to all future information supplied to you. This material may be disclosed to and used by your staff and associates as may be necessary to conduct the clinical study.

| **Version:** Final V1.1 | **Date of Version:** 8th December 2008 |
| --- | --- |

# TABLE OF CONTENTS

[**TABLE OF CONTENTS 2**](#__RefHeading___Toc201566023)

[SIGNATURE PAGE 8](#__RefHeading___Toc201566024)

[LIST OF ABBREVIATIONS 9](#__RefHeading___Toc201566025)

[PROTOCOL SYNOPSIS 11](#__RefHeading___Toc201566026)

[1. KEY ROLES AND FACILITIES 16](#__RefHeading___Toc201566027)

[1.1 Principal Investigator 16](#__RefHeading___Toc201566028)

[1.2 Study Location 16](#__RefHeading___Toc201566029)

[1.3 Study Sponsor 16](#__RefHeading___Toc201566030)

[1.4 Financial Sponsor 16](#__RefHeading___Toc201566031)

[1.5 Biostatistics 17](#__RefHeading___Toc201566032)

[2. INTRODUCTION 18](#__RefHeading___Toc201566033)

[2.1 Background 18](#__RefHeading___Toc201566034)

[2.1.1 Therapeutic Area Background 18](#__RefHeading___Toc201566035)

[2.1.2 Description of Study Agent 19](#__RefHeading___Toc201566036)

[2.1.3 Effect of SPL7013 Gel on Condom Integrity 20](#__RefHeading___Toc201566037)

[2.1.4 Mechanism of Action 20](#__RefHeading___Toc201566038)

[2.1.4.1 Anti-HSV Activity 21](#__RefHeading___Toc201566039)

[2.1.4.2 Anti-HIV-1 Activity 21](#__RefHeading___Toc201566040)

[2.1.4.3 Contraceptive Activity 22](#__RefHeading___Toc201566041)

[2.1.5 In Vitro Studies 23](#__RefHeading___Toc201566042)

[2.1.5.1 Cytotoxicity 23](#__RefHeading___Toc201566043)

[2.1.5.2 Spermatozoa Motility 23](#__RefHeading___Toc201566044)

[2.1.5.3 Genetic Toxicity 23](#__RefHeading___Toc201566045)

[2.1.6 Animal Studies 24](#__RefHeading___Toc201566046)

[2.1.6.1 Oral Administration 24](#__RefHeading___Toc201566047)

[2.1.6.2 Intravenous Administration 24](#__RefHeading___Toc201566048)

[2.1.6.3 Vaginal Administration 24](#__RefHeading___Toc201566049)

[2.1.6.4 Penile Administration 26](#__RefHeading___Toc201566050)

[2.1.6.5 Rectal Administration 26](#__RefHeading___Toc201566051)

[2.1.6.6 Developmental Toxicity 26](#__RefHeading___Toc201566052)

[2.1.6.7 Pharmacokinetics 27](#__RefHeading___Toc201566053)

[2.1.7 Summary of Clinical Studies 27](#__RefHeading___Toc201566054)

[2.1.7.1 SPL7013-001 27](#__RefHeading___Toc201566055)

[2.1.7.2 SPL7013-002 29](#__RefHeading___Toc201566056)

[2.1.7.3 SPL7013-004 (DMID 05-0121) 29](#__RefHeading___Toc201566057)

[2.1.7.4 SPL7013-006 (MTN-004) 30](#__RefHeading___Toc201566058)

[2.1.8 INSTEAD® SoftCup Sampling Method 31](#__RefHeading___Toc201566059)

[2.2 Rationale 32](#__RefHeading___Toc201566060)

[2.3 Limitations of Study Design 33](#__RefHeading___Toc201566061)

[2.4 Potential Risks and Benefits 34](#__RefHeading___Toc201566062)

[3. STUDY OBJECTIVES 34](#__RefHeading___Toc201566063)

[3.1 Primary Objective 34](#__RefHeading___Toc201566064)

[3.2 Secondary Objectives and Endpoints 34](#__RefHeading___Toc201566065)

[4. STUDY DESIGN 34](#__RefHeading___Toc201566066)

[5. STUDY POPULATION 34](#__RefHeading___Toc201566067)

[5.1 Target Population 34](#__RefHeading___Toc201566068)

[5.1.1 Inclusion Criteria 35](#__RefHeading___Toc201566069)

[5.1.2 Exclusion Criteria 35](#__RefHeading___Toc201566070)

[5.1.3 Randomisation Criteria 36](#__RefHeading___Toc201566071)

[5.2 Number of Subjects 36](#__RefHeading___Toc201566072)

[5.3 Method of Subject Assignment 36](#__RefHeading___Toc201566073)

[6. STUDY PRODUCT 37](#__RefHeading___Toc201566074)

[6.1 Investigational Product 37](#__RefHeading___Toc201566075)

[6.2 Dosage and Administration 37](#__RefHeading___Toc201566076)

[6.2.1 Dose and Dose Justification 37](#__RefHeading___Toc201566077)

[6.2.2 Administration 38](#__RefHeading___Toc201566078)

[6.3 Supply, Packaging, Labelling and Storage 38](#__RefHeading___Toc201566079)

[6.4 Treatment Allocation and Randomisation 39](#__RefHeading___Toc201566080)

[6.5 Dispensing and Accountability 39](#__RefHeading___Toc201566081)

[6.6 Assessment of Subject Compliance with Study Product 39](#__RefHeading___Toc201566082)

[6.7 Concomitant Medication/Treatment 39](#__RefHeading___Toc201566083)

[6.8 Precautions 40](#__RefHeading___Toc201566084)

[6.9 Warnings 40](#__RefHeading___Toc201566085)

[7. STUDY PROCEDURES AND EVALUATIONS 41](#__RefHeading___Toc201566086)

[7.1 Assessment Periods 41](#__RefHeading___Toc201566087)

[7.1.1 Screening Procedures 41](#__RefHeading___Toc201566088)

[7.1.2 Study Procedures 42](#__RefHeading___Toc201566089)

[7.1.2.1 Pre-dose sampling 42](#__RefHeading___Toc201566090)

[7.1.2.2 Treatment Period 1 43](#__RefHeading___Toc201566091)

[7.1.2.3 Treatment Periods 2-5 43](#__RefHeading___Toc201566092)

[7.1.2.4 Follow-up Visit 44](#__RefHeading___Toc201566093)

[7.2 Observation and Measurements: Assessment of Treatment Effects 44](#__RefHeading___Toc201566094)

[7.2.1 Cervico-vaginal Sampling 44](#__RefHeading___Toc201566095)

[7.2.2 Viral replication assays 45](#__RefHeading___Toc201566096)

[7.2.2.1 HIV Infectivity Assay 45](#__RefHeading___Toc201566097)

[7.2.2.2 HSV Infectivity Assay 46](#__RefHeading___Toc201566098)

[7.2.3 Pharmacokinetic Assay 46](#__RefHeading___Toc201566099)

[7.2.4 Handling and Processing of the Biological Specimens 46](#__RefHeading___Toc201566100)

[7.2.5 Storage and Destruction of the Biological Specimens 46](#__RefHeading___Toc201566101)

[7.3 Safety Measurements 46](#__RefHeading___Toc201566102)

[7.3.1 Physical Examination 46](#__RefHeading___Toc201566103)

[7.3.2 Vital Signs 46](#__RefHeading___Toc201566104)

[7.3.3 Laboratory Safety Testing 46](#__RefHeading___Toc201566105)

[7.3.3.1 Biochemistry 46](#__RefHeading___Toc201566106)

[7.3.3.2 Haematology 46](#__RefHeading___Toc201566107)

[7.3.3.3 Serology 47](#__RefHeading___Toc201566108)

[7.3.3.3.1 HIV testing 47](#__RefHeading___Toc201566109)

[7.3.3.3.2 Other Serology 48](#__RefHeading___Toc201566110)

[7.3.3.4 Urinalysis 48](#__RefHeading___Toc201566111)

[7.3.4 Adverse Events 48](#__RefHeading___Toc201566112)

[7.4 Study Restrictions 49](#__RefHeading___Toc201566113)

[7.4.1 Dietary 49](#__RefHeading___Toc201566114)

[7.4.2 Smoking 49](#__RefHeading___Toc201566115)

[7.4.3 Confinement 49](#__RefHeading___Toc201566116)

[7.4.4 Physical and Sexual Activity 49](#__RefHeading___Toc201566117)

[7.4.5 Concomitant Medication 49](#__RefHeading___Toc201566118)

[7.4.6 Environmental Conditions 50](#__RefHeading___Toc201566119)

[8. SAFETY REPORTING 50](#__RefHeading___Toc201566120)

[8.1 Adverse Event Definitions 50](#__RefHeading___Toc201566121)

[8.1.1 Adverse Event (AE) 50](#__RefHeading___Toc201566122)

[8.1.2 Serious Adverse Event (SAE) 51](#__RefHeading___Toc201566123)

[8.1.3 Unexpected Adverse Drug Reaction 52](#__RefHeading___Toc201566124)

[8.2 Reporting of an Adverse Event 52](#__RefHeading___Toc201566125)

[8.2.1 Adverse and/or Medically Significant Events 52](#__RefHeading___Toc201566126)

[8.2.2 Serious Adverse Event 52](#__RefHeading___Toc201566127)

[8.3 Recording of an Adverse Event 54](#__RefHeading___Toc201566128)

[8.4 Clinical Laboratory Abnormalities and Other Abnormal Assessments as Adverse Events or Serious Adverse Events 56](#__RefHeading___Toc201566129)

[8.5 Toxicity Management 56](#__RefHeading___Toc201566130)

[8.6 Follow-Up of Adverse Events 57](#__RefHeading___Toc201566131)

[8.7 Expedited Adverse Event Reporting Requirements 57](#__RefHeading___Toc201566132)

[8.7.1 Expedited Adverse Event Reporting to DAIDS and Starpharma Pty Ltd 57](#__RefHeading___Toc201566133)

[8.7.2 EAE Reporting Requirements for this Study 58](#__RefHeading___Toc201566134)

[8.8 Regulatory Reporting Requirements 58](#__RefHeading___Toc201566135)

[8.9 IEC/IRB Reporting Requirements 59](#__RefHeading___Toc201566136)

[8.10 Pregnancy 59](#__RefHeading___Toc201566137)

[9. CLINICAL MANAGEMENT 60](#__RefHeading___Toc201566138)

[9.1 Subject Completion 60](#__RefHeading___Toc201566139)

[9.2 Subject Withdrawal 60](#__RefHeading___Toc201566140)

[9.3 Procedures for Handling Withdrawals 60](#__RefHeading___Toc201566141)

[9.4 Replacement of Withdrawn Subjects 60](#__RefHeading___Toc201566142)

[9.5 Loss to Follow-Up 61](#__RefHeading___Toc201566143)

[9.6 Premature Termination of Study 61](#__RefHeading___Toc201566144)

[10. STATISTICS 61](#__RefHeading___Toc201566145)

[10.1 Criteria for Evaluation of Study Objectives 61](#__RefHeading___Toc201566146)

[10.1.1 Primary Endpoints 61](#__RefHeading___Toc201566147)

[10.1.2 Definition for Evaluation of Secondary/Safety Study Objectives 62](#__RefHeading___Toc201566148)

[10.1.2.1 Safety Parameters 62](#__RefHeading___Toc201566149)

[10.2 Description of Subject Groups for Analysis 62](#__RefHeading___Toc201566150)

[10.2.1 Definition of Subject Completion 62](#__RefHeading___Toc201566151)

[10.2.2 Definition of Datasets for Analysis 62](#__RefHeading___Toc201566152)

[10.2.2.1 Safety Dataset 62](#__RefHeading___Toc201566153)

[10.2.2.2 Per Protocol Dataset 62](#__RefHeading___Toc201566154)

[10.2.3 Datasets analysed for determination of study endpoints and analysis 62](#__RefHeading___Toc201566155)

[10.2.3.1 Primary Endpoints 62](#__RefHeading___Toc201566156)

[10.2.3.2 Safety Analysis 62](#__RefHeading___Toc201566157)

[10.3 Sample Size Estimation 62](#__RefHeading___Toc201566158)

[10.4 Statistical and Analytical Plan 63](#__RefHeading___Toc201566159)

[10.5 Analysis of Demographics 63](#__RefHeading___Toc201566160)

[10.6 Analysis of Safety 63](#__RefHeading___Toc201566161)

[10.7 Interim Analysis 63](#__RefHeading___Toc201566162)

[11. HUMAN SUBJECTS PROTECTION 63](#__RefHeading___Toc201566163)

[11.1 Regulatory Considerations 63](#__RefHeading___Toc201566164)

[11.2 Independent Ethics Committee (IEC)/Institutional Review Board (IRB) 64](#__RefHeading___Toc201566165)

[11.3 Interpretation of the Protocol / Protocol Amendments 64](#__RefHeading___Toc201566166)

[11.4 Subject Informed Consent 64](#__RefHeading___Toc201566167)

[11.5 Confidentiality 65](#__RefHeading___Toc201566168)

[12. ADMINISTRATIVE ASPECTS 65](#__RefHeading___Toc201566169)

[12.1 Clinical Trial Agreement 65](#__RefHeading___Toc201566170)

[12.2 Study File 66](#__RefHeading___Toc201566171)

[12.3 Initiation of the Study 66](#__RefHeading___Toc201566172)

[12.4 Subject Reimbursement 66](#__RefHeading___Toc201566173)

[12.5 Subject Identification 66](#__RefHeading___Toc201566174)

[12.6 Confidential Follow-up 66](#__RefHeading___Toc201566175)

[12.7 Recording of Data 67](#__RefHeading___Toc201566176)

[12.8 Monitoring of the Study 67](#__RefHeading___Toc201566177)

[12.9 Protocol Deviations 68](#__RefHeading___Toc201566178)

[12.10 Data Quality Control 69](#__RefHeading___Toc201566179)

[12.11 Quality Assurance Audit/Inspection 69](#__RefHeading___Toc201566180)

[12.12 Study and Site Closure 69](#__RefHeading___Toc201566181)

[12.13 Record Retention 70](#__RefHeading___Toc201566182)

[12.14 Study Report 70](#__RefHeading___Toc201566183)

[13. INDEMNITY AND COMPENSATION 71](#__RefHeading___Toc201566184)

[13.1 Insurance 71](#__RefHeading___Toc201566185)

[14. SPONSOR RESPONSIBILITIES 71](#__RefHeading___Toc201566186)

[14.1 Funding 71](#__RefHeading___Toc201566187)

[14.2 Supply of Study Materials and Study Documentation 71](#__RefHeading___Toc201566188)

[14.3 Compliance with Regulatory Requirements 71](#__RefHeading___Toc201566189)

[14.4 Transfer of Sponsor Obligations 71](#__RefHeading___Toc201566190)

[15. USE OF DATA AND PUBLICATIONS 72](#__RefHeading___Toc201566191)

[16. APPENDICES 72](#__RefHeading___Toc201566192)

[16.1 References 72](#__RefHeading___Toc201566193)

[16.2 Declaration of Helsinki 74](#__RefHeading___Toc201566194)

[16.3 Outcomes, Diagnostics and Follow-Up Evaluations 75](#__RefHeading___Toc201566195)

# SIGNATURE PAGE

The signatures below constitute approval of this protocol and the attachments, and provide the necessary assurances that this trial will be conducted according to all stipulations of the protocol, including all statements regarding confidentiality, and local legal and regulatory requirements including ICH guidelines.

Dr Peter Hodsman Date

Principal Investigator, Nucleus Network

Peter Mullins Date

Biostatistician, Trident Clinical Research

Jeremy Paull, PhD Date

VP, Development and Regulatory Affairs

Starpharma

Clare Price Date

Clinical Development Manager

Starpharma

# LIST OF ABBREVIATIONS

| AE | Adverse Event / Adverse Experience |
| --- | --- |
| AIDS | Acquired Immunodeficiency Syndrome |
| ALP | Alkaline Phosphatase |
| ALT | Alanine Aminotransferase |
| APTT | Activated Partial Thromboplastin Time |
| ASCUS | Atypical Squamous cells of Undetermined Significance |
| AST | Aspartate Transaminase |
| BMI | Body Mass Index |
| BUN | Blood Urea Nitrogen |
| CBC | Complete Blood Count |
| CFR | Code of Federal Regulations |
| CRF | Case Report Form |
| CIOMS | Council for International Organizations of Medical Sciences |
| CONSORT | Consolidated Standards of Reporting Trials |
| CRF | Case Report Form |
| CRO | Contract Research Organization |
| CV | Cervico-vaginal |
| DCC | Data Coordinating Centre |
| DLT | Dose Limiting Toxicity |
| DSMB | Data Safety Monitoring Board |
| DSMC | Data Safety Monitoring Committee |
| ECG | Electrocardiogram |
| FBE | Full Blood Examination |
| FDA | Food and Drug Administration |
| GCP | Good Clinical Practice |
| GLP | Good Laboratory Practice |
| GMP | Good Manufacturing Practice |
| HCV | Hepatitis C Virus |
| HIV | Human Immunodeficiency Virus |
| HPV | Human Papilloma Virus |
| HSV-2 | Herpes Simplex Virus Type 2 |
| IB | Investigator’s Brochure |
|  |  |
| ICF | Informed Consent Form |
| ICH | International Conference on Harmonization |
| IDE | Investigational Device Exemption |
| IDT | Institute of Drug Technology |
| IEC | Independent or Institutional Ethics Committee |
| IND | Investigational New Drug |
| IRB | Institutional Review Board |
| ISM | Independent Safety Monitor |
| KOH | Potassium Hydroxide |
| MCH | Mean Corpuscular Haemoglobin |
| MCHC | Mean Corpuscular Haemoglobin Concentration |
| MCV | Mean Cell Volume |
| MDR | Maximum Dose Reached |
| MED | Minimum Effective Dose |
| MedDRA | Medical Dictionary for Regulatory Activities |
| MOP | Manual of Procedures |
| MTD | Maximum Tolerated Dose |
| N | Number (typically refers to subjects) |
| PCR | Polymerase Chain Reaction |
| PI | Principal Investigator |
| PK | Pharmacokinetics |
| PT | Prothrombin Time |
| QA | Quality Assurance |
| QC | Quality Control |
| RBC | Red Blood Cells |
| SAE | Serious Adverse Event |
| SHIV | Simian Human Immunodeficiency Virus |
| SMC | Safety Monitoring Committee |
| SOP | Standard Operating Procedure |
| Starpharma | Starpharma Pty Ltd |
| STD | Sexually Transmitted Disease |
| STI | Sexually Transmitted Infection |
| TPPA | Treponema Palladium Particle Agglutination |
| UNAIDS | Joint United Nations Programme on HIV/AIDS |
| USA | United States of America |
| WBC | White Blood Cells |
| WHO | World Health Organization |

# PROTOCOL SYNOPSIS

| **Study Title:** | Assessment of local retention and duration of activity of SPL7013 following vaginal application of 3% SPL7013 Gel (VivaGel®) in healthy volunteers |
| --- | --- |
| **Short Title:** | Retention and duration of activity of SPL7013 after vaginal dosing |
| **Sponsor:** | Starpharma Pty Ltd |
| **Protocol Number:** | SPL7013-003 |
| **Development Phase:** | Phase 1 |
| **Proposed Indication:** | Prevention of HIV and HSV infection in women |
| **Primary Objective:** | The primary objective of the study is to assess the local retention and antiviral activity of SPL7013 in cervico-vaginal (CV) samples as a function of time after application of 3% SPL7013 Gel in healthy volunteers. |
| **Secondary Objective(s):** | The secondary objective of the study is to assess safety, including local tolerability and systemic safety. |
| **Hypothesis:** | The hypothesis is that SPL7013 remains bioavailable and antiviral activity is retained for a period of time after application. |
| **Primary Endpoints:** | Ex vivo activity in the inhibition of virus replication as measured in *ex vivo* replication assays for HIV and HSV using CV samples, and mass and concentration of SPL7013 determined from the CV contents taken at screening, 0[[1]](#footnote-2), 1, 3, 12 and 24 hours after vaginal application of 3% SPL7013 Gel and a further sample at follow-up visit. |
| **Secondary Endpoints:** | None. |
| **Safety Endpoints:** | Local and systemic toxicity |
| **Study Design:** | Single-centre, open-label, randomised, cross-over, Phase 1 study. Each subject will receive 5 single doses of 3% SPL7013 Gel with at least 5 days washout between doses. One CV sample will be taken after each dose application at screening and at 0, 1, 3, 12 and 24 hours after dosing in a randomised sequence. Three additional CV samples will be taken pre-dose for use in validation and as standards in the activity and content assays and a further sample at the follow-up visit. |
| **Sample Size:** | 12 healthy women |
| **Summary Inclusion Criteria:** | - Female volunteers aged 18 to 45 years inclusive with regular menstrual cycles and predictable menses - Body mass index (BMI) between 18 and 30 kg/m^2^ - Healthy as assessed by medical history and physical examination - Negative pregnancy test at screening and baseline and at each study visit - Negative result or completed evaluation of atypical squamous cells of undetermined significance (ASCUS) on Pap smear documented at or within 12 months before screening. - Has provided written informed consent to participate in the study. - Able to comply with study procedures and follow instructions from staff. - Contraception: Must use lubricated (non-Nonoxynol-9 [N-9]) condoms plus one other acceptable form of contraception; i.e. sterilisation (for more than 3 months), intra-uterine device (inserted at least 3 months prior to enrolment), or hormonal contraception (except inter-vaginal products). - Able to abstain from sexual intercourse (including penile-vaginal and oral-vaginal) according to the study restrictions (i.e. refrain from intercourse between study treatment administration and CV sampling, and for 24 hours before study treatment administration or administration of the screening/pre-dosing CV samples. |
| **Summary Exclusion Criteria:** | - History or presence of clinically relevant autoimmune, cardiovascular, pulmonary, gastrointestinal, hepatic, renal, metabolic, haematological, neurological, psychiatric, systemic or infectious disease, any acute infectious disease or signs of acute illness. - Abnormal pelvic examination, including presence of genital warts or pre-cancerous lesions, which in the Investigator’s opinion indicates the woman is unsuitable for the study. - Presence or history of allergy to topical vaginal products including any known hypersensitivity to the components of SPL7013 Gel, or to latex condoms. - Confirmed positive reaction to the following tests: hepatitis B surface antigen, anti-HCV antibodies, anti-HIV-1 antibodies, anti-HIV2 antibodies. - Participant has received an investigational drug within 30 days or 5 half-lives, whichever is the longer, prior to entering the study. - History of recurrent vaginal infections, irritation or localised reactions to vaginally applied agents. - Current urinary tract infection (UTI) - Participant tests positive for an STI during screening evaluations (Chlamydia, gonorrhoea or HSV-2), or has been treated for an STI during the three months prior to enrolment. - Unpredictable or irregular menstrual cycle that will not allow scheduling of the CV sampling outside menstruation as required for the protocol. This would include an intramenstrual period les than 16 days. - Recent history of intramenstrual bleeding (but heavier than spotting) - Use of intravaginal preparations within 14 days prior to drug administration or during the study. - Pregnancy or breast-feeding - Menopause - Hysterectomy or recent gynaecological surgery (within 3 months of screening) - Vaginitis or vaginosis (symptomatic) - Participant, in the opinion of the investigator, should not participate in the study |
| **Study Duration and Duration/Subject:** | Total duration of approximately 13 weeks, comprising 8 weeks on study plus up to 4 weeks screening and one week follow-up |
| **Study Product:** | 3.5g of 3% *w/w* SPL7013 Gel (VivaGel®) |
| **Study Procedures:** | After a 4-week screening period, each subject will receive 5 single doses of 3% SPL7013 Gel with at least 5 days washout between doses. CV samples will be taken using an INSTEAD® SoftCup at screening and at 0, 1, 3, 12 and 24 hours after dosing in a randomised sequence. Three additional blank samples will be taken at pre-dose and a further sample at the follow-up visit. |
| **Lab Analyses:** | The following assays will be conducted on the CV samples:   - Cell culture-based assays to determine the levels of the anti-HIV and anti-herpes simplex virus (HSV) activity of SPL7013 in CV samples - Bioanalytical assay for concentration of SPL7013 |
| **Safety Parameters:** | Routine biochemistry, haematology and urinalysis, physical examination/medical history, adverse events. |
| **Statistical Analyses:** | 12 subjects have been chosen for this initial evaluation of the bioavailability and antiviral activity of SPL7013 in CV samples over time after vaginal application of 3% SPL7013 Gel. No formal statistical determination has been performed.  For the primary endpoints of prevention of HIV and HSV ex vivo, the antiviral activity will be measured as a percent inhibition of replication of each virus (% inhibition).The level of inhibition from samples taken immediately after product application (time=0) will be the Baseline reference value (100% inhibition) for each subject. The level of inhibition at all other time points will be expressed as % inhibition relative to the level of inhibition at Baseline. The comparison of mean % inhibition will be performed for each time point versus that at Baseline using an appropriate statistical test to determine if there is a statistically significant decrease in activity compared to 0 hours. If there is a statistically significant difference between the values at each time point and the 0 hour time point, comparisons may be made between the other time points.  The concentration and mass of SPL7013 in CV samples at each time point (mass is calculated from the measured concentration of SPL7013 in each CV sample) will be compared to the values immediately after application using an appropriate statistical test as outlined below. If possible (i.e. the semi-log plot of concentration and/or mass vs time is linear), an estimate of the half-life of SPL7013 concentration and/or mass in CV contents will be calculated. |

**Table 1 Schedule of Study Assessments**

|  | **Screening** | **Pre-dose** | **Treatment**[[2]](#footnote-3) | | | | | **Follow-up** |
| --- | --- | --- | --- | --- | --- | --- | --- | --- |
| Procedure |  | CV samples[[3]](#footnote-4) | Period 1 | Period 2 | Period 3 | Period 4 | Period 5 |  |
| Informed Consent | x |  |  |  |  |  |  |  |
| Inclusion/exclusion criteria | x |  |  |  |  |  |  |  |
| Medical/Surgical History | x |  |  |  |  |  |  |  |
| Serology[[4]](#footnote-5) | x |  |  |  |  |  |  | x |
| Pregnancy test | x |  | x | x | x | x | x | x |
| Pap smear[[5]](#footnote-6) | x |  |  |  |  |  |  |  |
| Height and weight | x |  |  |  |  |  |  |  |
| Physical examination[[6]](#footnote-7) | x |  |  |  |  |  |  | x |
| Visit to unit | x |  | x | x | x | x | x | x |
| Randomization |  |  | x |  |  |  |  |  |
| Study drug administration |  |  | x | x | x | x | x |  |
| ENDPOINTS |  |  |  |  |  |  |  |  |
| Vaginal sampling[[7]](#footnote-8) | x | x | x | x | x | x | x | x |
| SAFETY |  |  |  |  |  |  |  |  |
| Vital signs | x |  |  |  |  |  |  | x |
| Haematology and Biochemistry | x |  |  |  |  |  |  | x |
| Urinalysis | x |  |  |  |  |  |  | x |
| AE collection | x[[8]](#footnote-9) | x | x | x | x | x | x | x |
| Prior/Concomitant Meds | x | x | x | x | x | x | x | x |

# KEY ROLES AND FACILITIES

## Principal Investigator

Dr Peter Hodsman

Medical Director

Nucleus Network Ltd

5th Floor, Burnet Tower

89 Commercial Road

Melbourne Victoria 3004

Australia

## Study Location

Nucleus Network Ltd

5th Floor, Burnet Tower

89 Commercial Road

Melbourne Victoria 3004

Australia

Tel: +61 3 9076 8892

Fax: +61 3 9076 8911

## Study Sponsor

Starpharma Pty Ltd

Baker Building

75 Commercial Road

Melbourne Victoria 3004

Australia

Tel: +61 3 8532 2700

Fax: +61 3 9510 5955

## Financial Sponsor

DAIDS, NIAID, NIH

Roberta Black, PhD

Chief, Microbicide Research Branch

Jeanna Piper, MD

Senior Medical Officer

Microbicide Research Branch

Prevention Sciences Program

6700B Rockledge Drive

Bethesda, MD 20892-7628, USA

Tel: +1 301 496 8199 / +1 301 451 2778

Fax: +1 301 402 3684

Contract No. HHSN266200500042C, ADB Contract No. N01-AI-50042

## Biostatistics

Peter Mullins

Trident Clinical Research

G05, 12-14 Cato Street

Hawthorn East Victoria 3123

Australia

Tel: +61 3 8317 5300

Fax: +61 3 9822 7581

# INTRODUCTION

## Background

## Therapeutic Area Background

SPL7013 Gel (VivaGel®) is being developed as a microbicide for the prevention of the sexual transmission of human immunodeficiency virus (HIV) and herpes simplex virus type 2 (HSV-2) infections.

According to the Joint United Nations Programme on HIV/AIDS (UNAIDS) and World Health Organization (WHO), every day, over 6800 people around the world become infected with HIV and over 5700 people died from AIDS in 2007. Sub-Saharan Africa remains the most seriously affected region, with AIDS remaining the leading cause of death there. An estimated 2.1 million deaths worldwide were due to AIDS, of which 76% occurred in sub-Saharan Africa, where heterosexual transmission is by far the most prominent mode of transmission of HIV. Almost 61% of adults living with HIV in this geographical region were women.^[1]^

Genital herpes, caused by HSV-2, is one of the most common sexually transmitted infections (STIs). In the United States (US), one in five teenagers and adults is infected with HSV-2, while one in four women are infected. Genital herpes is characterized by high rates of clinical and sub clinical reactivation in the genital mucosa and the associated risk of sexual transmission. Symptoms of the disease include sores, or ulcers, in and around the vaginal area and within the cervix in women, and on the penis and scrotum in men. Both symptomatic and asymptomatic recurrence of the disease can result in sexual transmission. Studies have shown that HSV-2 is the most common cause of genital ulcers in both developed and developing regions of the world and a meta-analysis of the risk of HIV infection in HSV-2 seropositive persons showed that HSV-2 infection is a significant risk factor for HIV acquisition.^[2]^

There is a need for new technologies to prevent the sexual transmission of HIV-1 and other pathogens such as genital HSV-2. Despite years of effort, effective preventive vaccines remain elusive.^[3]^ Despite the availability of male condoms, women are often unable to negotiate the use of condoms by their male partners. The female condom has recently been marketed as an alternative barrier method, but use of this device requires a certain level of skill, and at least the consent of the male partner.^[4,5]^

Topical microbicides are products that are designed to inhibit the sexual transmission of HIV and other pathogens.^[4,5,6,7]^ Microbicides could potentially be applied vaginally to prevent both male-to-female and female-to-male sexual transmission of HIV and other pathogens. Starpharma is initially focusing on prevention of male-to-female transmission. Microbicides also offer a female-controlled prophylactic option in cases in which male condom use cannot be negotiated.

A vaginal microbicide gel is expected to provide coverage for the female during sexual intercourse that is similar to that provided by the male condom, which has been shown to offer significant protection against HSV-2 infection in susceptible women.^[8]^

Given the possible modes of transmission and the viral replication cycles of HIV and HSV, it is hypothesised that optimal topical microbicides should block attachment or fusion of the virus to the cell membrane and/or interrupt viral replication prior to reverse transcription or integration. In developing a preventive strategy, targeting these early infection events seems a most promising mode of action.

This study is part of a development program designed to evaluate SPL7013 Gel as a candidate microbicide for the prevention of HIV transmission. Under the NIH-funded HIV Microbicide Design and Development Teams contract (Contract No. HHSN266200500042C), clinical safety data will be generated to support an efficacy evaluation of SPL7013 Gel in women.

## Description of Study Agent

REMOVED – PROPRIETARY INFORMATION

## INSTEAD® SoftCup Sampling Method

The INSTEAD® SoftCup is a soft disposable cup, made from polyethylene and mineral oil (see Figure 1). When inserted correctly, the cup sits under the cervix and behind the pubic bone, and a sample of cervicovaginal contents remains in the cup when it is removed.

Figure 1 INSTEAD® SoftCup


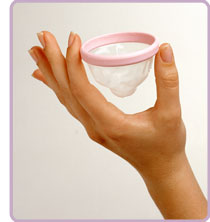


Cervicovaginal sampling via the INSTEAD® SoftCup method has been selected as it is less invasive for the study participant than cervicovaginal lavage, and avoids the unknown amount of dilution that is a factor when using cervicovaginal lavage for this type of study.

A pilot study to investigate sampling of cervicovaginal secretions found the method rapid and convenient for obtaining relatively large volumes of undiluted secretions.^[24]^ Pilot studies conducted at Starpharma indicate that employing a method of spinning down the SoftCup with contents in an adapted Falcon tube, after adding a set amount of saline solution, provides a recovery of between 98-103% of gel when at least 75 mg (2% of full dose) of gel is present.

It is not expected that the materials of the INSTEAD® SoftCup would interfere with the infectivity and pharmacokinetic assays as the cup is made from polyethylene, which is inert in biological fluids. In addition, samples will not be exposed to extremes of temperature. Since the gel is being extracted from the cup with dilute saline it is considered that there is a low probability of extractable components from the cup in the samples for analysis.  In the unlikely event of there being an extractable component present (e.g. plasticizers), the pharmacokinetic analysis relies on the compounds of interest (i.e. SPL7013) being (a) negatively charged at pH ~ 9.1 and (b) having UV absorbance at 240 nm. The chances of any extracted components from the cup having the right ionization and UV detection properties to interfere with the pharmacokinetic assay are very small.

Each study subject will be trained in the insertion and removal of the cup as part of the screening process, and a female member of the study site staff will check that the training has been successful.

## Rationale

Microbicides intended for the prevention of HIV or genital herpes are being advanced into late stage clinical studies based on antiviral activity observed *in vitro* and in animal models, and on clinical and non-clinical safety. While not intended as a definitive determinant of clinical efficacy, the current study will provide initial data on the activity of 3% SPL7013 Gel in cervico-vaginal (CV) samples after vaginal application of the product in humans. This clinical study will also be the first to determine the pharmacokinetics of SPL7013 from cervicovaginal samples, with all other clinical pharmacokinetic assessments having been undertaken with serum samples.

For future clinical studies in healthy volunteers at risk of HIV or HSV infection, knowledge of the time period during which SPL7013 remains bioavailable and antiviral activity is retained after application of SPL7013 Gel will enable guidance to be provided to participants on the time before intercourse that the gel should be applied. The current study is designed to provide data to support these instructions.

With this objective in mind, the study has been designed such that each woman acts as her own control. It is not the intention of this study to investigate the discrete activity of each component of the product mix *in vivo* at a given time-point (i.e. active, vehicle and CV fluid), and therefore no placebo arm has been included.

Time-points of 0, 1, 3, 12 and 24 were chosen for the practical reasons and to provide an indication of the half life of the product, and a 5-day wash-out between applications was considered by the study team to be sufficient time for washout of the gel to occur based on unpublished data from investigation of another microbicide.

## Limitations of Study Design

It is acknowledged that CV fluid in itself has intrinsic antiviral properties, and that the amount, constituency and corresponding antiviral properties of the CV fluid will vary with the menstrual cycle. The volume and visco-elasticity of CV fluid tend to increase during the follicular development and decrease in the immediate postovulatory phase of the menstrual cycle ^[21]^.

The variation in antiviral properties is considered to be minimal in comparison with those of the active product, and therefore it is not expected to confound study results. Sampling at certain points within the menstrual cycle would lead to significant complexity within the study, and as variability is considered minimal, has not been considered necessary to build in to this study design. A baseline sample will provide an indication of the intrinsic antiviral activity of CV fluid in each woman.

The variation in volume throughout the menstrual cycle may lead to variation in the dilution of the SPL7013 samples taken throughout the study. The literature indicates a mean increase in daily CV fluid volume (±SD) from 0.1 (±0.01)mL on day -6 to 0.7 (±0.40)mL on day -1 followed by a decrease to 0.6 (±0.40)mL on day 0 ^[22]^, providing a mean variation of approximately 0.69mL. In the context of administration of a 3.5g application of SPL7013 Gel, this variation translates to an increase in percentage CV fluid in the CV fluid / SPL7013 Gel mixture in the vagina, from approximately 3% to 17%. This degree of variation is not considered significant within the context of the objective of the study which is to understand when a gross drop in SPL7013 and antiviral activity may occur, in order to provide an indication of dosing timings for future efficacy studies. At worst, the results will provide an under-estimate of activity and amount of SPL7013 remaining.

Magnetic resonance imaging techniques have demonstrated that coating of the vagina and cervix with microbicide gels reduces with time over the 24 hour period, with varying distribution ^[23]^. It is also accepted that due to the anatomy of the vaginal vault and sampling method employed, an unknown proportion of the gel might not be accessed by the INSTEAD SoftCup. Both these factors may lead to an under-estimate of the activity and amount of SPL7013 remaining.

In addition, It is also acknowledged that full mixing of the CV fluid and SPL7013 Gel within the vaginal vault may not occur under the circumstances of the study, however as part of the sample processing procedures, the sample retrieved using the INSTEAD SoftCup is to be vortexed, which will result in sufficient mixing of all components for antiviral testing and accurate determination of SPL7013 concentration in the sample. The concentration of SPL7013 will be used to determine the amount of SPL7013 by taking in to account the weight of the sample obtained.

## Potential Risks and Benefits

There will be no direct benefit to study participants enrolled in this study, however the data produced may benefit future populations in directing product use to prevent infection with HIV or HSV-2.

It is anticipated that there will be minimal risks to study participants. There may be mild vaginal or cervical irritation associated with insertion and removal of the INSTEAD® SoftCup and slight discomfort associated with the drawing of blood samples. In addition, there may be effects as detailed in Section 6.9.

# STUDY OBJECTIVES

## Primary Objective

The primary objective of the study is to evaluate the local retention and duration of antiviral activity of SPL7013 in CV samples collected using the INSTEAD® SoftCup as a function of time after vaginal application of 3% SPL7013 Gel in healthy volunteers. The antiviral activity will be assessed using *ex vivo* assays for HIV and HSV replication. The amount of SPL7013 will be determined by measuring the concentration in CV contents using a validated assay.

## Secondary Objectives and Endpoints

The secondary objective is to assess safety, including local tolerability and systemic safety, as determined by incidence of AEs, vital signs and laboratory abnormalities.

# STUDY DESIGN

This is a single-centre, randomised, multiple-dose, open-label, 5-way cross-over study. Study participants will undertake five single applications of 3% SPL7013 Gel, each dose separated by a minimum of 5 days. CV samples will be taken at 0, 1, 3, 12 and 24 hours post-application in a randomised fashion and a further sample at the follow-up visit. The study is expected to be approximately 13 weeks in duration per participant. This comprises; a 4 week screening period; a 3 day pre-dose CV sampling period; a dosing and sampling period of approximately 8 weeks spanning 2 menstrual cycles; and a follow-up period of approximately 1 week following last sample.

# STUDY POPULATION

To assess any potential impact on subject eligibility with regard to safety, the investigator must refer to the Clinical Investigators’ Brochure (CIB)^[11]^ for detailed information regarding warnings, precautions, contraindications, adverse events, and other significant data pertaining to 3% SPL7013 Gel.

## Target Population

The target population for recruitment is healthy female volunteers, aged 18 to 45 years.

## Inclusion Criteria

1. Female volunteers aged 18 to 45 years inclusive with regular menstrual cycles and predictable menses.
2. Body mass index (BMI) between 18 and 30 kg/m^2^.
3. Healthy as assessed by medical history and physical examination.
4. Negative pregnancy test at screening and at baseline and at each study visit.
5. Negative result or completed evaluation of atypical squamous cells of undetermined significance (ASCUS) on Pap smear documented at or within 12 months before screening.
6. Has provided written informed consent to participate in the study.
7. Able to comply with study procedures and follow instructions from staff.
8. Contraception: Must use lubricated (non-Nonoxynol-9 [N-9]) condoms plus one other acceptable form of contraception; i.e. sterilisation (for more than 3 months), intra-uterine device (inserted at least 3 months prior to enrolment), or hormonal contraception (except inter-vaginal products).
9. Able to abstain from sexual intercourse (including penile-vaginal and oral-vaginal) according to the study restrictions (i.e. refrain from intercourse between study treatment administration and CV sampling, and for 24 hours before study treatment administration or administration of the screening and pre-dosing CV samples).

## Exclusion Criteria

1. History or presence of clinically relevant autoimmune, cardiovascular, pulmonary, gastrointestinal, hepatic, renal, metabolic, haematological, neurological, psychiatric, systemic, or infectious disease; any acute infectious disease or signs of acute illness.
2. Abnormal pelvic examination, including presence of genital warts or pre-cancerous lesions, which in the Investigator’s opinion indicates the woman is unsuitable for the study.
3. Presence or history of allergy to topical vaginal products including any known hypersensitivity to the components of SPL7013 Gel, or to latex condoms.
4. Confirmed positive reaction to the following tests: hepatitis B surface antigen, anti-HCV antibodies, anti-HIV-1 antibodies, anti-HIV-2 antibodies.
5. Participant has received an investigational drug with 30 days or 5 half-lives, whichever is the longer, prior to entering the study.
6. History of recurrent vaginal infections, irritation or localised reaction to vaginally applied agents.
7. Current urinary tract infection (UTI).
8. Participant tests positive for an STI during screening evaluations (Chlamydia, gonorrhoea or HSV-2), or has been treated for an STI during the three months prior to enrolment.
9. Unpredictable or irregular menstrual cycle that will not allow scheduling of the CV sampling outside menstruation as required for the protocol. This would include an intramenstrual period less than 16 days.
10. Recent history of intramenstrual bleeding (but heavier than spotting).
11. Use of intravaginal preparation within 14 days prior to drug administration or during the study.
12. Pregnancy or breast-feeding.
13. Menopause.
14. Hysterectomy or recent gynaecological surgery (within 3 months of screening).
15. Vaginitis or vaginosis (symptomatic).
16. Participant, in the opinion of the investigator, should not participate in the study.

## Randomisation Criteria

All inclusion and exclusion criteria must be met before any subject can be randomised into the study.

## Number of Subjects

A total of 12 eligible subjects are to be enrolled into the study.

Drop-outs (as defined in Section 9) can be replaced at the discretion of the Sponsor.

## Method of Subject Assignment

This is an open-label study. Study participants will be allocated a unique participant identification number at randomization in sequential order.

# STUDY PRODUCT

## Investigational Product

## Dosage and Administration

REMOVED – PROPRIETARY INFORMATION

## Supply, Packaging, Labelling and Storage

Product labelling should comply with Australian Therapeutics Goods Administration (TGA) and US Food and Drug Administration (FDA) requirements for investigational products.

Each applicator will be over-wrapped in a sealed opaque envelope, and the overwrap will be labelled to comply with TGA and FDA requirements as above.

## Treatment Allocation and Randomisation

Participants will be randomly allocated to one of the following study groups. Their sampling sequence is determined by the group to which they are randomised.

|  | **Treatment Period 1** | **Treatment Period 2** | **Treatment Period 3** | **Treatment Period 4** | **Treatment Period 5** |
| --- | --- | --- | --- | --- | --- |
| **Group A** | 0 h | 1 h | 3 h | 12 h | 24 h |
| **Group B** | 1 h | 3 h | 12 h | 24 h | 0 h |
| **Group C** | 3 h | 12 h | 24 h | 0 h | 1 h |
| **Group D** | 12 h | 24 h | 0 h | 1 h | 3 h |
| **Group E** | 24 h | 0 h | 1 h | 3 h | 12 h |

## Dispensing and Accountability

Study product is to be stored in a locked cupboard at the site, or within the pharmacy. Drug is to be requested via a product request form or prescription which is to be signed and dated by the Principal Investigator, Co- or Sub-Investigator(s). A Pharmacy Establishment Plan will be prepared for the study.

The Principal Investigator will retain accurate records of receipt of all test articles, including dates of receipt. In addition, accurate records will be kept regarding when and how much of each test article was administered to each individual subject in the study.

Reasons for departure from the expected dispensing regimen must also be recorded. At completion of the study all study medication must be reconciled via detailed records itemizing all movement of test article to, from and within study site, and dispensing records.

## Assessment of Subject Compliance with Study Product

Study participants will be issued each applicator and instructed to insert the gel in a private room whilst at the study site, under supervision of the study staff. A subject will be considered to be compliant if she has administered all 5 doses (for those subjects who complete the study), or all doses up to withdrawal (for those subjects who prematurely withdraw).

## Concomitant Medication/Treatment

Vaginal preparations including lubricants, anti-fungals and antibiotics, will be not be allowed during the study. Lubricated (non-N-9 containing) condoms will be supplied to the participants, to be used during the periods of the study where sexual activity is permissible.

There are no additional restrictions on concomitant medications administered during the study, however all medications must be reported and recorded in the CRF (to include generic or trade name for products with one active ingredient, or trade name for combination products, dose, form and duration of treatment). If the concomitant medication is required as a treatment for an adverse event, then this must also be recorded in the CRF.

## Precautions

The Principal Investigator, Co- and Sub-Investigators and all study staff must familiarize themselves with the pre-clinical and clinical data to date as outlined in the IB.

## Warnings

Based on the animal pharmacokinetic and toxicology data and the clinical data from the first human study, the following events may be experienced following application of SPL7013 Gel:

1. vaginal and cervical irritation;
2. vaginal itching;
3. pain with urination;
4. abdominal pain or discomfort;
5. vaginal discharge of a curdled white material;
6. changes in vaginal microflora.

Although not expected, other risks that are currently unknown may be associated with the treatment. These will be fully documented as the studies progress.

Systemic absorption of SPL7013 following vaginal administration of SPL7013 containing gels has not been observed in either animal or human studies.

The reproductive effects of SPL7013 Gel have not been investigated in clinical trials. The effects of SPL7013 Gel on the developing human foetus and breast-feeding child are also unknown. For this reason, women of childbearing potential must agree to use adequate contraception (i.e. hormonal contraceptive or IUD, or sterilization) prior to study entry and for the duration of the study. Healthy women recruited in to this study will undertake a pregnancy test prior to receiving each dose of SPL7013 gel. Should a woman become pregnant or suspects that she is pregnant while participating in the study, she should inform study staff and her primary care physician immediately. Women found to be pregnant will not receive any further doses of study product but every attempt will be made by study staff to follow the participant for pregnancy outcome. In addition, women who are breast-feeding are not to be enrolled in to the study.

As SPL7013 Gel is an investigational product; it has not yet been shown in clinical trials to reduce the risk of HSV-2 or HIV infection or any other sexually transmitted disease. It is therefore essential that in addition to other contraceptive methods, a condom is used with each coital act to minimize the risk of sexually transmitted infections.

# STUDY PROCEDURES AND EVALUATIONS

This is a single-centre, open-label, randomised, cross-over, Phase 1 study.

After providing one screening and three pre-dose cervico-vaginal (CV) samples, each subject will receive 5 single doses of 3% SPL7013 Gel with at least 5 days washout between doses. CV samples will be taken at 0 (between 2 and 10 minutes), 1, 3, 12 and 24 hours after dosing in a randomised sequence and a further sample at the follow-up visit as shown in Figure 2.

All study visits will be scheduled such that they do not fall within 2 days before and 5 days after the expected onset of menses. Product administration and CV sampling is not to take place during menstruation.

## Assessment Periods

Figure 2 Study Flow Chart

## Screening Procedures

Screening procedures will be carried out up to 28 days prior to enrolment. Each volunteer will receive information on the study design and procedures and will have to sign and date the informed consent before any study procedures are performed. If necessary, a study participant may stay overnight at the clinic in order to ensure all screening procedures are performed adequately.

The screening visit will include the following procedures:

- Sign and date informed consent
- Previous medical and surgical history
- Medical examination, height, weight and BMI
- Physical examination including pelvic examination
- Prior and concomitant medications
- Vital sign measurements
- Blood and urine sampling for laboratory determination (biochemistry, haematology, serology and HIV status (with information on the testing and counselling, STI evaluation and urinalysis - see Section 7.3.3 for full details)
- Pregnancy testing
- Screening signs and symptoms
- Review of inclusion/exclusion criteria
- Pap smear (if no adequate result obtained in past year)
- Demonstration of study drug administration (using empty applicator)
- Condom and sexual restrictions counselling (see Section 7.4.4)
- Demonstration and training in CV sampling method using the INSTEAD SoftCup (see Section 7.2.1)
- Collection of CV sample

Volunteers who meet all the inclusion/exclusion criteria will be eligible for enrolment.

## Study Procedures

## Pre-dose sampling

Following enrolment the participants will be given three CV sampling kits and asked to provide three separate samples from three separate days. The samples should be taken at least 24 hours apart during the intra menstrual period (i.e. should not include menses). The participants will not be required to attend the study centre and will be able to send the samples into the clinical unit by courier, however if they experience difficulty undertaking the sampling, they may attend the clinical unit and be assisted by female staff. The courier will be in the form of a taxi service, regularly used by the clinical study site for this function, and the sample will be stored in refrigerated conditions on ice packs in an opaque, insulated container during transit. The sample will be marked as an uninfected biological sample; however the nature of the sample will not be identifiable to the courier to protect the privacy of the study subject. The sample is to be transported to the study centre within 24 hours of collection, if not; the sample is to be repeated.

During this period, any AEs reported will be recorded as screening/baseline signs and symptoms, and medications recorded as concomitant medication.

## Treatment Period 1

The first treatment period will include the following procedures:

- Review of the inclusion/exclusion criteria
- Review of screening/baseline signs and symptoms pre-dose and AEs post dose and concomitant medications
- Pregnancy test
- Randomisation to a sampling sequence
- Condom and sexual restrictions counselling (see Section 7.4.4)
- Administration of 3% SPL7013 Gel by study participant. If the study participant is not considered to be able to administer the product correctly after training during screening, or is uncomfortable doing so, a female member of the study team may administer the product.
- CV sampling using the INSTEAD SoftCup at 0, 1, 3, 12 or 24 h post application according to the randomization schedule. A time window of within 5 minutes is permissible for the 0 and 1 hour time points, and within +/-10% of the time for the 3, 12 and 24 hour time points (see Section 7.2.1)

Study participants will be permitted to leave the clinical unit between study treatment administration and CV sampling, except when a time 0 or 1 hour sample is to be collected. If necessary, study participants may stay overnight at the clinic.

## Treatment Periods 2-5

Treatment periods 2–5 will include the following procedures:

- Review of AEs and concomitant medications
- Review of study exclusions and restrictions
- Pregnancy test
- Condom and sexual restrictions counselling (see Section 7.4.4)
- Administration of 3% SPL7013 Gel
- CV sampling using the INSTEAD SoftCup at 0, 1, 3, 12 or 24 h post application according to the randomisation schedule (see Section 7.2.1).

Study participants will be permitted to leave the clinical unit between study treatment administration and CV sampling, except when a time 0 or 1 hour sample is to be collected. If necessary, study participants may stay overnight at the clinic.

## Follow-up Visit

All participants will be invited for a follow-up visit at least 1 week after the final study treatment administration. This visit will include the following procedures:

- Review of AEs and concomitant medications
- Review of study exclusions and restrictions
- Physical examination including pelvic examination
- Pregnancy test
- Vital sign measurements
- Blood and urine sampling for laboratory determination (biochemistry, haematology, serology and urinalysis)
- Final CV sample

## Observation and Measurements: Assessment of Treatment Effects

## Cervico-vaginal Sampling

CV contents will be collected at screening, pre-dose and at 0, 1, 3, 12 and 24 hours post treatment application and a further sample at the follow-up visit. CV contents will be sampled only once per treatment application. Therefore, each participant will receive 5 treatments. Participants will be randomised to a specific sampling sequence upon enrolment.

Each participant will collect their own CV contents samples using the INSTEAD® SoftCup. This sampling method has been chosen as it allows sampling over a large area, which is comparable to cervical lavage, and allows collection of undiluted samples therefore avoiding the unknown degree of dilution seen with cervical lavage.^[24]^ In addition, the cup is simple to use and requires minimal training, which is ideal for women who are unfamiliar with the device. Using the SoftCup for collection is less invasive than lavage as it can be easily inserted and removed by study participants in the privacy of a bathroom cubicle.

Participants will be provided with both written and video instructions as per the INSTEAD® SoftCup website [http://softcup.com/product/softcup_directions.php, and <http://softcup.com/product/video.php>]. They will be trained in the use of the INSTEAD® SoftCup at the screening visit, when at least one screening sample will be taken. If the participant experiences difficulties in insertion, a study Investigator may aid in the insertion or removal of the SoftCup.

Approximately 15 minutes prior to the sampling time-point, study participants will be directed to the bathroom with an INSTEAD® device in its factory over-wrap, and a tube in an opaque bag. The weight of the tube and the wrapped INSTEAD® will have been pre-recorded. Participants will be instructed to unwrap, place the wrapper back in the bag and insert the INSTEAD® cup until it is fully inside the vagina with the rim tucked up behind the pubic bone, as per the INSTEAD® instructions. Participants will be instructed to remove the cup after approximately 10-30 seconds (i.e. the SoftCup is to remain in the vagina for 10-30 seconds for each CV sample), and insert into the tube provided, recap the tube, and place the tube into the bag. The opaque bag will then be handed to the study staff.

Three baseline samples will also be taken during the pre-dose period for assay validations. The same sampling method will be used except that the participants will be able to carry out this sampling at home (see Section 7.1.2.1). They will be provided with sampling kits (INSTEAD® SoftCup, centrifuge tube, sampling form, and opaque paper bag) during the screening visit and will be able to send the samples to the clinical unit by courier. As above, if any participant experiences difficulties in insertion, she may attend the clinical unit and a study Investigator may aid in the insertion or removal of the SoftCup. The three samples should be taken on different days at least 24 hours apart, and will be kept refrigerated until transportation by the courier. These samples are needed for use in validation and as standards in the activity and content assays.

The exact weight of the sample of gel plus vaginal secretions can then be calculated by subtracting the pre-recorded weight of the empty cup from the final weight of the filled cup. The centrifuge tube containing the INSTEAD® SoftCup and collected samples will be stored at 4^o^C and the gel processed and recovered from the tube within 24 hours.

The samples will be processed to remove the gel and samples, aliquoted and then stored frozen at -80 ^o^C until analysis for SPL7013 concentration and activity using the assays described below.

The handling, processing, recovery and storage of CV samples is described in detail in the sample collection, processing and recovery standard operating procedures.

## Viral replication assays

## HIV Infectivity Assay

The anti-HIV activity of SPL7013 present in CV samples will be mainly determined against a HIV-1 strains that utilises the CCR5 chemokine coreceptor for viral entry which is the most relevant with respect to the sexual transmission of HIV-1.^[27]^ Assays will be performed in the TZM-bl indicator cell line using luciferase as the readout for HIV replication.^[28,29]^ All samples will be tested against HIV_Ba-L_ a clade B laboratory strain which grows to high titres. Clade B is the major circulating strain of HIV-1 in the USA and Australia.

## HSV Infectivity Assay

The anti-HSV activity of SPL7013 in CV samples will be determined for HSV type 2 in HEL cells using cell viability as the readout for viral replication.^[30]^ The HSV isolate that will be used in the assay is a highly cytopathic clinical isolates obtained from Australian patients and isolated and typed by the Victorian Infectious Diseases Reference Laboratory (VIDRL).

## Pharmacokinetic Assay

CV samples will be assayed for SPL7013 levels using a validated capillary electrophoresis bioanalytical method at the Starpharma Pty Ltd bioanalytical laboratory, Melbourne, Australia.

## Handling and Processing of the Biological Specimens

The handling, processing, recovery and storage of CV samples is described in detail in the sample collection, processing and recovery standard operating procedures.

## Storage and Destruction of the Biological Specimens

After processing, CV samples will be stored in frozen conditions (-80^o^C). These samples will be stored indefinitely for potential future assays.

## Safety Measurements

## Physical Examination

Each subject will undergo a physical examination to include a pelvic/gynaecological examination. All conditions identified at screening will be recorded in the Medical History section of the case report form (CRF).

## Vital Signs

Heart rate and systolic and diastolic blood pressure will be measured after 5 minutes in a sitting position.

## Laboratory Safety Testing

## Biochemistry

The following biochemistry parameters will be assessed: Plasma electrolytes (sodium, potassium, and calcium); liver function (AST, ALT, alkaline phosphatase, gamma-GT, total and conjugated bilirubin); metabolism (fasting glucose, albumin, total cholesterol, triglycerides); renal function (creatinine).

## Haematology

The following haematological parameters will be assessed: Haemoglobin (Hb); white blood cell count (WBC) with differential (neutrophils, eosinophils, basophils, monocytes and lymphocytes); platelets.

## Serology

## HIV testing

Detection of HIV-1 and/or HIV-2 antibodies will be undertaken using a chemiluminescent microparticle immunoassay. Information and counselling will be provided both pre- and post-testing.

The following algorithm will be employed in order to interpret the results:

**A) INTERPRETATION OF RESULTS**

- Specimens with S/CO values between 0.9 and 0.99 are considered grey zone reactive. These samples are to be treated as potentially positive and are to be followed up as for a positive sample.
- Specimens with S/CO values >1.0 are considered reactive by the criteria of the ARCHITECT HIV-Ag/Ab Combo assay.
- Specimens with S/CO values <0.9 are considered negative by the criteria of ARCHITECT HIV-Ag/Ab assay.

All initially reactive specimens should be retested in duplicate after centrifugation at 10,000 g for 10 minutes in a microcentrifuge.

Aliquots for retesting MUST be taken from the initial aliquot taken off for storage purposes. If on retesting the specimen is reactive it is considered to be repeatedly reactive by the criteria of ARCHITECT HIV Ag/Ab assay.

Repeatedly reactive specimens need to have a HIV western blot performed at VIDRL **PRIOR to any result being reported.**

**Only those specimens found reactive by western blot are considered positive for antibodies to HIV-1 and/or HIV-2.**

Samples found HIV antibody **negative** at VIDRL will have a HIV p24 performed to exclude its presence.

**NOTE: If, upon retesting, an initially reactive sample is non reactive, two consistent negative results must be obtained PRIOR to reporting the result as negative. If either of the retest values is reactive, the sample is considered to be repeatedly reactive and a HIV western blot must be performed PRIOR to any result being reported.**

Interpretation of specimens found repeatedly reactive by the ARCHITECT HIV Ag/Ab Combo assay and non-reactive or indeterminate by supplemental tests for either HIV-1 and/or HIV-2 antibody is unclear. The presence of HIV p24 antigen must be excluded if supplemental tests for HIV-1 and/or HIV-2 antibody are not reactive. Early in seroconversion illness HIV p24 antigen may be present prior to antibody production.

Further clarification of antibody status may be obtained by testing another specimen taken three to six weeks

Collection of a blood tube containing acid dextrose (ACD) may be collected for referral to NRL for a proviral DNA test if clinically appropriate. This test will detect patients in the early seroconversion stage of the illness.

**B) REPORTING**

Non reactive specimens are reported as negative.

- Repeatedly reactive specimens are referred to VIDRL for confirmatory testing (HIV Western Blot) PRIOR to any report being released on the computer. A verbal report is given to Dr. Denis Spelman or one of the senior infectious diseases physicians.
- Repeatedly reactive specimens, which are also reactive on the HIV western blot, are reported as positive. Notify the pathology office when a positive or indeterminate report is verified.
- Pathology office staff should deliver the report to serology.
- Serology staff place the result in a sealed white envelope addressed to the referring doctor and marked **PRIVATE AND CONFIDENTIAL.**

## Other Serology

The following serological evaluations will be performed:

- Hepatitis B antigen - HBsAg plus confirmatory test if positive - Architect,  Anti-HBsAnti-HBc (total) - Architect, Anti-HBcIgM - Vidas (enzyme-linked fluorescence assay)
- HCV - Anti- HCV Architect, Murex Anti-HCV for positives (supplementary testing).
- HSV by ELISA, with PCR confirmation.

Information and counselling will be provided both pre- and post-testing.

## Urinalysis

A standard dipstick urinalysis will be performed including leukocytes.

In addition, a PCR will be performed for *N. gonorrhoeae* and *C. trachomatis.*

## Adverse Events

The Investigator and designated study personnel will monitor each subject for AEs during the study. All AEs reported between consent and final follow-up will be recorded in the CRF. The investigator or designee will ask the subject non-leading questions in an effort to detect AEs. Examples of such questions are:

*“How are you feeling?”*

Or

*“Since you were last asked, have you felt unwell or different from usual?”*

In addition, subjects should be encouraged to spontaneously report any unusual feelings or sensations. See Section 8. for full details on adverse experience reporting.

## Study Restrictions

## Dietary

There are no dietary restrictions during the study.

## Smoking

Participants must not smoke when visiting the study unit. There are no restrictions on smoking outside the unit.

## Confinement

Participants will be allowed to leave the clinical unit between study treatment administration and CV sampling, except when a time 0 sample or 1 hour sample is to be collected. If necessary, study participants may stay overnight at the clinic.

## Physical and Sexual Activity

The participants will be asked to refrain from intensive physical exercise and penile-vaginal and oral-vaginal intercourse between study treatment administration and CV sampling and for 24 hours before study treatment administration or before the pre-dose samples are taken. They will be allowed and encouraged to ambulate normally.

## Concomitant Medication

Vaginal preparations including lubricants will be not be allowed during the study. Lubricated condoms (non-N-9 containing) will be supplied to the participants. If the study participant develops a vaginal infection (other than an STI) after enrolment in to the study for which vaginally administered products are indicated and prescribed (i.e. to treat symptomatic candidiasis or symptomatic bacterial vaginosis), study visits will be suspended until at least 5 days after both the symptoms are completely resolved (as determined by the Principal Investigator), and the vaginally administered product is no longer being used.

## Use of Tampons and Sanitary Pads

Tampons are not to be inserted within 48 hours after application of study product, but may be used at any other time. If sanitary protection is required during the restricted period, then sanitary pads are to be used. Sanitary pads will be provided to study participants if requested, and panty liners to protect against gel leakage.

## Environmental Conditions

Except for the smoking restrictions outlined above, there are no restrictions on the environmental conditions under which CV samples and other study assessments are to be undertaken.

# SAFETY REPORTING

The reporting and documentation of AEs and clinically significant laboratory abnormalities or other assessments is an essential component of all clinical studies. Therefore, it is important that all investigational staff understand the requirements and responsibilities outlined below. It is the responsibility of the Investigator to ensure that all AEs and other clinically significant findings that occur during the conduct of a clinical study are documented and reported accurately.

AE(s) should be documented in terms of a medical diagnosis(ies), rather than signs and/or symptoms, where possible.

## Adverse Event Definitions

## Adverse Event (AE)

At each evaluation the investigator will determine whether any AEs have occurred by asking a non-leading question.

An AE includes any untoward medical occurrence in a patient or clinical investigational subject administered an investigational product (whether it is the experimental product or the control) and which does not necessarily have a causal relationship to the Study drug (whether it is the experimental product or the control).

The investigator will make a judgment regarding whether or not, in their opinion, the adverse event was related to the test drug. However, even if the investigator feels there is no relationship to the test drug, the AE should be reported. If any clinical adverse events have occurred they will be recorded on the AE report page of the CRF and their intensity will be graded.

AEs may include:

1. The significant worsening of the disease or symptoms of the disease under investigation following administration of investigational product/drug.
2. An intercurrent illnesses with an onset after administration of investigational product/drug.
3. Exacerbation (i.e., increase in frequency or intensity) of a pre-existing condition or event.

An AE does not include a/an:

1. Medical or surgical procedure: the condition that leads to the procedure is an AE.
2. Situations where an untoward medical occurrence has not occurred (e.g. hospitalization for cosmetic/elective surgery, social and/or convenience admissions).
3. Overdose of either study drug or concomitant medication that does not result in any signs or symptoms. (Note: Australian TGA requires Overdose reported as an SAE). If any signs or symptoms of an overdose present, then these will be recorded as an AE

## Serious Adverse Event (SAE)

An SAE is defined as any adverse drug experience occurring at any dose that results in any of the following outcomes:

- Results in death^b^
- Is life-threatening^c^
- Requires in-patient hospitalization or prolongation of existing hospitalization
- Results in persistent or significant disability / incapacity
- Is a congenital anomaly / birth defect

^a^ **Occurring at any dose**: Does not imply that the subject is receiving study drug at the time of the event. Dosing may have been given as treatment cycles or interrupted temporarily prior to the onset of the SAE, but may have contributed to the event.

^b^ **Death**: Death is an outcome of an SAE, and not an SAE in itself. All deaths must be reported for volunteers on study and for deaths occurring within 30 days of last study drug dose or within 30 days of last study evaluation, whichever is longer, to Starpharma Regulatory and QA Department, who in turn will ensure immediate (with 24 hours) reporting to DAIDS Medical Officer, and reporting to TGA and FDA within the required timelines. The investigator should supply Starpharma and the IEC / IRB with any additional requested information as available (e.g. autopsy reports and terminal medical reports). Study participants will be asked to carry a card for 30 days post last study evaluation to identify that they have participated in a study, and provide contact details in the event of death.

^c^ **Life-threatening**: The term “life-threatening” in the definition of “serious” refers to an event in which the subject was at immediate risk of death at the time of the event. It does not refer to an event which hypothetically might have caused death if it were more serious.

^d^ **Hospitalization**: This is defined as the subject being hospitalized overnight, or the subject’s hospital stay being prolonged for at least an additional overnight stay. Hospital admissions for a pre-existing condition (e.g. elective surgery) or for normal disease management procedures (e.g. chemotherapy) will not be considered as serious. Complications that occur during hospitalizations are AEs. If a complication prolongs hospitalization, it is an SAE.

Important medical events that may not result in death, be life-threatening, or require hospitalization may be considered a serious adverse event when, based upon appropriate medical judgment, they may jeopardize the study participant and may require medical or surgical intervention to prevent one of the outcomes listed in this definition. Examples of such medical events include allergic bronchospasm requiring intensive treatment in an emergency room or at home, blood dyscrasias or convulsions that do not result in inpatient hospitalization, or the development of drug dependency or drug abuse.

.

## Unexpected Adverse Drug Reaction

Unexpected Adverse Drug Reaction is defined as an adverse reaction to study drug that is not identified in nature, severity or frequency in the IB or that is not expected from the characteristics of study drug.

## Reporting of an Adverse Event

## Adverse and/or Medically Significant Events

AE reporting will begin from first dose and will continue throughout the study until the Exit Evaluation.

## Serious Adverse Event

SAE reporting will begin from first dose and will continue until the Exit Evaluation.

**ANY** **SERIOUS ADVERSE EVENT** (INCLUDING DEATH) **DUE TO ANY CAUSE** WHICH OCCURS DURING THE COURSE OF THE INVESTIGATION, WHETHER OR NOT RELATED TO THE STUDY DRUG (INVESTIGATIONAL DRUG), **MUST BE REPORTED IMMEDIATELY** (within 24 hours of the investigator becoming aware of the event) TO STARPHARMA REGULATORY & QA DEPARTMENT AS FOLLOWS:

**Starpharma Switchboard Number: +61 3 8532 2700**

**Starpharma Fax Number: +61 3 9510 5955**

Telephone contact must be made with either the sponsor or study monitor in order to make an initial SAE report. Specific contact details and 24-hour contact details are available in the Study Procedures Manual.

Starpharma is responsible for reporting all SAEs to the DAIDS Medical Officer immediately upon being notified (within 24 hours).

The investigator (or delegate) will be requested to complete the Starpharma SAE form including as much information regarding the event that is available at the time of the initial report.

Investigators should not wait to receive additional information to fully document the event before notifying Starpharma of an SAE.

Prompt notification is essential so that legal requirements and ethical obligations to the subjects participating in the study can be met. A telephone report must be followed by a written report including copies of relevant medical records, autopsy reports and other relevant documents.

When reporting SAEs, the investigator should not include the name or address of the individual subject.

Minimum requirements for SAE reporting:

- Protocol number
- Centre name/ID
- Reporting investigator’s/physician’s name
- Subject initials, randomization number, age and sex
- Study drug dose and duration of treatment
- Condition treated (medical diagnosis, if known)
- Nature and severity of the SAE
- Relationship of SAE to Study drug
- Potentially confounding factors/concomitant medication*
- Action taken/outcome*

* Where action was taken or concomitant medication was administered, ensure that corresponding CRF pages have been completed.

The investigator must also:

- Report all SAEs to the reviewing IEC / IRB within the time-line specified by the reviewing body;
- Submit follow-up reports to Starpharma until the SAE has resolved or, in the case of permanent impairment, until the SAE stabilizes.

If the SAE is study drug related and unexpected, the SAE will be reported by Starpharma to the FDA, TGA and DAIDS Medical Officer within required timeframes (i.e. within 7 (fatal or life-threatening) or 15 (non-fatal or not life-threatening) calendar days of the event occurring and/or becoming aware of the event).

## Recording of an Adverse Event

- Adverse events All adverse events are to be recorded on the ‘Adverse Event’ pages within the subject’s CRF.
- Serious Adverse Events In addition to the ‘Adverse Event’ page, SAEs are to be recorded on the Starpharma Serious Adverse Event reporting form and then a copy is inserted into the study subject’s CRF.

At each clinical evaluation the investigator/delegate will determine whether any adverse events have occurred. The subject will be questioned in a non-leading way and no specific symptoms will be suggested. If known, the medical diagnosis of an AE should be recorded in preference to the listing of individual signs and symptoms.

The investigator/delegate will make a judgment regarding whether or not, in his/her opinion the study drug had any possible causal relationship to the Adverse Event. The investigator/delegate will evaluate any changes in laboratory values; make a determination as to whether or not the change is clinically important, and whether or not the changes were related to the study drug. However, even if the investigator/delegate feels there is no relationship to the study drug, the adverse event must be recorded in the CRF.

If any adverse events are present when a subject completes the study or when a subject is withdrawn from the study, the investigator/delegate should make every effort to follow-up the subject until the adverse event has resolved or stabilized. All follow-up information (and attempted follow-up contacts) should be documented in the subject’s medical records.

The severity of adverse events is graded in the first instance according to the Division of AIDS Female Genital Grading Table for Use in Micribocide Studies available on the RCC website at: http://rcc.tech-res.com. Those events not listed in the Female Genital Grading Table will be graded according to The Division of AIDS Table for Grading the Severity of Adult and Pediatric Adverse Events (DAIDS AE Grading Table), Version 1.0, December, 2004, also available at http://rcc.tech-res.com.

Those adverse events not covered within either of the above toxicity tables will be graded as follows:

***Grade 1: Mild*** An AE that causes no or minimal interference with usual social and functional activities.

***Grade 2: Moderate*** An AE that causes greater than minimal interference with usual social and functional activities.

***Grade 3: Severe*** An AE that causes inability to perform usual social and functional activities.

***Grade 4: Life Threatening*** An AE that causes inability to perform basic self-care functions or medical or operative intervention is indicated to prevent permanent impairment, persistent disability or death.

***Grade 5: Death*** An AE that results in death.

It should be noted that an AE that is considered to be “severe” may not necessarily be considered to be “serious” or of major medical significance.

The relationship to study drug therapy should be assessed using the following definitions:

***Not Related*** The adverse event is clearly explained by another cause not related to the study agent.

***Probably Not Related*** A potential relationship between study agent and the adverse event could exist (i.e. the possibility cannot be excluded), but the adverse event is most likely explained by causes other than the study agent.

***Possibly Related*** The adverse event and administration of study agent are reasonably related in time, and the adverse event can be explained equally well by causes other than the study agent.

***Probably Related*** The adverse event and administration of study agent are reasonably related in time, and the adverse event is more likely explained by study agent than other causes.

***Definitely Related*** The adverse event and administration of study agent are related in time, and a direct association can be demonstrated.

It should be emphasized that ineffective treatment should not be considered as causally related in the context of AE reporting.

These criteria in addition to good clinical judgment should be used as a guide for determining the causal assessment.

The degree of certainty with which an adverse event is attributed to study drug/product treatment or alternative cause, (e.g. natural history of the underlying diseases, concomitant therapy, etc) will be determined by how well the event can be understood in terms of:

- Known pharmacology of the product.
- Reaction of a similar nature previously observed with similar products/drugs.
- The event having often been reported in literature for similar products as product related.
- The event being related by time to product/drug administration/ termination, product/drug withdrawal, or reproduced on rechallenge.

## Clinical Laboratory Abnormalities and Other Abnormal Assessments as Adverse Events or Serious Adverse Events

Laboratory abnormalities deemed not clinically significant and not considered an AE according to the DAIDS toxicity tables are usually not recorded as AEs or SAEs. However, all abnormal laboratory findings (e.g. clinical chemistry, haematology, urinalysis) or other abnormal assessments (e.g. vital signs) that meet the definition (or cause signs and symptoms that meet the definition) of an AE (or SAE) must be reported as outlined above.

## Toxicity Management

AEs and laboratory abnormalities will be graded as outlined in Section 8 .

For the purpose of monitoring toxicities, the baseline value is defined as the last value prior to the administration of the first dose of study medication. All management is based on changes from this value.

All grade 2 or higher laboratory abnormalities should be confirmed by repeat testing as soon as possible, preferably within 3 calendar days of receipt of results prior to dose interruption or discontinuation, unless such a delay is not consistent with good medical practice.

In response to AEs reported by study participants and/or observed upon exam by study staff, the study site investigator or designee will recommend either continuation or holding study gel use consistent with the criteria in Section 16.3. Study gel use also will be held or discontinued in the event of an expedited adverse event (EAE) that is judged by the site Investigator or designee to have a potential causal relationship with study product.

Unless the participant withdraws consent, she will remain in the study to complete the safety evaluations (unless clinically contraindicated according to Section 16.3).

Study participants who develop an RTI (i.e. bacterial vaginosis (BV) or candidiasis) requiring treatment whilst on study will not be withdrawn, but study product will be suspended until symptoms have resolved and treatment is completed as outlined in Section 7.4.5. However, study participants who develop an STI during the study period are to be withdrawn from the study.

## Pausing / Stopping Rules

The study will be paused if:

- 2 or more participants report a grade 3 or higher genital AE, considered to be related to study medication or
- Ulceration considered to be related to study medication and with no clear explaination is seen in 2 or more participants or
- study product is discontinued in 3 or more participants due to genital events considered to be related to study medication

In the event that a decision is taken to pause the study according to the above criteria, a full review will take place of all the AE data for all subjects by the Principal Investigator, DAIDS Medical Officer, Medical Monitor and Starpharma. The ethics committee and DAIDS are to be immediately notified of this decision. By reviewing the full data along with the data emerging from the ongoing study(ies) and that from existing studies, the team will come to a decision based on their judgement and expertise as to the next course of action, which could be:

1. To restart the study with no amendment to the protocol
2. To restart the study after implementation of a protocol amendment.
3. To place the study on clinical hold pending the emergence of further data.
4. To terminate the study

The ethics committee, TGA, FDA, and DAIDS are to be notified of any protocol amendment, study hold, or termination of the study according to applicable regulations as outlined in Sections 9.6 and 11.

## Follow-Up of Adverse Events

Investigators must follow up subjects with serious adverse events until the event has stabilised or resolved, whichever is the latter. In the case of non-serious adverse events, follow-up should occur until the subject completes the study, and if possible, until the event has stabilized or resolved. Records of the subject’s progress should be maintained until the conclusion of the study and thereafter in a subject’s medical history at the relevant investigational site. Details of the subject’s progress must be submitted to the Starpharma Study Monitor on request.

## Expedited Adverse Event Reporting Requirements

This section outlines Expedited Adverse Event (EAE) reporting requirements for this study. Study sites will receive training on EAE reporting prior to the onset of study enrolment.

## Expedited Adverse Event Reporting to DAIDS and Starpharma Pty Ltd

The EAE reporting requirements and definitions for this study and the methods for expedited reporting of AEs to the DAIDS RCC Safety Office are defined in “The Manual for Expedited Reporting of Adverse Events to DAIDS” (DAIDS EAE Manual) dated May 6, 2004. The DAIDS EAE Manual is available on the RCC website: <http://rcc.tech-res-intl.com/>. The DAIDS EAE Manual is also available in the Study Procedures Manual.

AEs reported on an expedited basis must be documented on the DAIDS Expedited Adverse Event Reporting Form (EAE Reporting Form) available on the RCC website: [http://rcc.tech-res-intl.com](http://rcc.tech-res-intl.com/).

EAEs must be faxed to DAIDS and Starpharma Pty Ltd as outlined in the Study Procedures Manual. The DAIDS Medical Officer is to receive timely and synchronous communications of any AE reported to the RCC.

## EAE Reporting Requirements for this Study

EAE Reporting Level

This study uses the Intensive Level of EAE reporting as defined in the DAIDS EAE Manual.

Study Agents for Expedited Reporting to DAIDS

The study agents that must be considered in determining relationships of AEs requiring expedited reporting to DAIDS are: 3% SPL7013 Gel, applicator and INSTEAD SoftCup.

Study Agents for Expedited Reporting to Starpharma Pty Ltd

The study agents that must be considered in determining relationships of AEs requiring expedited reporting to Starpharma Pty Ltd are: 3% SPL7013 Gel.

Grading Severity of Events

The severity of adverse events will be graded according to Section 8.3.

EAE Reporting Periods

AEs must be reported on an expedited basis at the Intensive Level during the Protocol-defined EAE Reporting Period, which is:

The entire study duration for an individual participant (from study enrolment until study completion or discontinuation of the participant from study participation for any reason).

After the end of the Protocol-defined EAE Reporting Period stated above, the site must report serious, unexpected, clinical suspected adverse drug reactions if the study site staff becomes aware of the event on a passive basis, i.e., from publicly available information.

## Regulatory Reporting Requirements

Starpharma has a legal responsibility to notify both the local regulatory authority (ies) (i.e. the TGA) and other overseas agencies (i.e. the FDA) about the safety of the product/drug under clinical investigation. Prompt notification of SAEs by the Investigator is essential so that legal obligations and ethical responsibilities towards the safety of other subjects are met.

## IEC/IRB Reporting Requirements

The time frame within which the Investigators must be notified of deaths, study drug related and/or unexpected SAEs are stipulated by the local regulatory authorities.

It is the Investigator’s responsibility to comply with the requirements for IEC/IRB notification. The Investigator will notify the IEC/IRB, by promptly forwarding the Safety Report completed by Starpharma or its agent, for all onward reportable SAEs.

## Pregnancy

Study participants who become pregnant during the study period (up to and including 30 days after the last dose of investigational product) must not receive additional doses of investigational product but may continue other study procedures at the discretion of the investigator.

Study participants should be instructed to notify the Investigator if after completion of the study they become pregnant whether during the treatment phase or within 30 days following the last administration of study product.

If any female subject becomes pregnant while enrolled in the clinical study and after receipt of study product, Starpharma must be notified within 24 hours of the Investigator learning of the pregnancy. The Investigator will be asked to complete the SAE CRF without the ‘criteria of seriousness’ checked.

If the subject is in the treatment phase of the study, she will be withdrawn from the clinical study and the procedure for discontinuation of a subject will be followed (see Section 9.3).

Whenever possible, a pregnancy in subjects exposed to study product should be followed to term. Any premature terminations and the status of the mother and child after delivery should be reported to Starpharma. The Pregnancy Outcome and Questionnaire Form may be used by the Investigator to record details of the pregnancy outcome. The form includes provision to record the mothers’ status during the pregnancy, relevant medical history, and details of the health of the baby(ies) on delivery.

If a congenital anomaly/birth defect occurs, this must be reported as a SAE.

# CLINICAL MANAGEMENT

## Subject Completion

A subject will be deemed to have completed the study once all trial procedures have been conducted. Any AEs or SAEs still ongoing will be followed in accordance with Section 8.

## Subject Withdrawal

The investigator should make every reasonable effort to keep each subject in the study, except where termination or withdrawal is for safety reasons. However, if the investigator removes a subject from the study, or the subject declines further study participation, a complete exit evaluation must be performed. Should a subject decide to withdraw from the study, all efforts will be made to complete and report the reason(s) for withdrawal and observations as thoroughly as possible.

The investigator may cease study treatment and withdraw the subject, or the subject may withdraw himself from participation in the study at any time and for any reason. The following are considered justifiable reasons for subject withdrawal from the study:

- The need to take medication which may interfere with study measurements;
- Intolerable/unacceptable adverse experiences;
- Major violation or deviation of study protocol;
- Non-compliance of subject with protocol;
- Subject unwilling to proceed and/or consent is withdrawn;
- Withdrawal from the study is, in the investigator’s judgment, in the subject’s best interest;
- Other (e.g. personal reasons).

## Procedures for Handling Withdrawals

The Investigator (or delegate) must complete the reason for withdrawal and the date of withdrawal of a subject in the appropriate section of the subject’s CRF. If more than one reason applies, then the main reason for withdrawal should be indicated.

In the event that a female subject becomes pregnant at any time during the study period every attempt will be made to follow her to term (although study participant has the option to decline further follow-up), the outcome of the mother and infant documented as per Section 8.11.

## Replacement of Withdrawn Subjects

Any subjects who discontinue the clinical study of their own volition or by the investigator are defined as “drop-outs”. Drop-outs can be replaced at the discretion of the Sponsor either before or after randomisation.

.

## Loss to Follow-Up

At the start of the study the volunteer will state how they can be contacted during the study. This will be recorded in the participants' address file. If a participant fails to appear for a scheduled visit, at least three attempts to contact them should be made over the subsequent 7 days. These attempts should be documented in the participant’s study file. After these three attempts, no further efforts need be made to find them, but their file should remain open until study closeout.

If the participant does not return to the clinic before the study is closed, the CRF should be checked to indicate that the participant was lost to follow-up. The “loss to follow-up” designation cannot be made for any participant until the closing date of the study.

## Premature Termination of Study

The study may be terminated prematurely by the principal investigator or his/her designee, the sponsor, DAIDS, the FDA or TGA if:

- The number and/or severity of adverse events justify discontinuation of the study
- New data become available which raise concern about the safety of the study drug, so that continuation might cause unacceptable risks to subjects.

In addition, Starpharma reserves the right to discontinue the trial prior to inclusion of the intended number of subjects, but intends only to exercise this right for valid scientific or administrative reasons.

After such a decision, the Investigator must contact all participating subjects within two weeks, and written notification must be sent to the Ethics Committee.

# STATISTICS

## Criteria for Evaluation of Study Objectives

## Primary Endpoints

The endpoints for evaluation of primary study objectives (Primary Endpoints) are as follows:

- The total amount (mass) of SPL7013 in CV contents sampled at 0, 1, 3, 12 and 24 hours after application.
- The concentration of SPL7013 in CV contents sampled at 0, 1, 3, 12 and 24 hours after application.
- The ex vivo activity in the prevention of replication of HIV of CV contents sampled at screening and at 0, 1, 3, 12 and 24 hours after application.
- The ex vivo activity in the prevention of replication of HSV of CV contents sampled at screening and at 0, 1, 3, 12 and 24 hours after application.

## Definition for Evaluation of Secondary/Safety Study Objectives

## Safety Parameters

The safety objectives of the study will be evaluated from the following parameters (Safety Parameters):

- Incidence of AEs.
- Haematology, biochemistry, vital signs

## Description of Subject Groups for Analysis

## Definition of Subject Completion

A subject is considered to have completed the study if they have completed all study visits, including all cervico-vaginal sampling procedures and the follow-up visit.

## Definition of Datasets for Analysis

## Safety Dataset

The Safety Dataset includes all subjects who were randomised in to the study and received at least one application of study medication, whether they dropped out during the study or not.

## Per Protocol Dataset

The Per Protocol Dataset includes all subjects who are randomised in to the study, and completed at least two treatment periods including the 0 h sampling time point and do not have any major protocol violations.

## Datasets analysed for determination of study endpoints and analysis

## Primary Endpoints

The primary endpoints will be determined from the Per Protocol dataset.

## Safety Analysis

The Safety analysis will be conducted using the Safety dataset.

## Sample Size Estimation

Twelve participants have been chosen for an initial evaluation of the retention of SPL7013 amount and antiviral activity over time after application of SPL7013 Gel. No formal statistical determination has been performed as these parameters have not been previously assessed and therefore no information exists about the variability of these endpoints. The number of study participants has been chosen as a reasonable number for an initial assessment of the primary endpoints.

## Statistical and Analytical Plan

For the primary endpoints of prevention of HIV and HSV ex vivo, the antiviral activity will be measured as a percent inhibition of replication of each virus (% inhibition) . The level of inhibition from samples taken immediately after product application (time=0) will be the Baseline reference value (100% inhibition) for each subject. The level of inhibition at all other time points will be expressed as % inhibition relative to the level of inhibition at Baseline. The comparison of mean % inhibition will be performed for each time point versus that at Baseline using an appropriate statistical test to determine if there is a statistically significant decrease in activity compared to 0 hours. If there is a statistically significant difference between the values at each time point and the 0 hour time point, comparisons may be made between the other time points.

The mass and concentration of SPL7013 in CV samples at each time point (mass is calculated from the measured concentration of SPL7013 in each CV sample) will be compared to the values immediately after application using an appropriate statistical test as outlined below. If possible (i.e. the semi-log plot of mass or concentration vs time is linear), an estimate of the half-life of SPL7013 mass and/or concentration in CV contents will be calculated.

Should the data be reasonably symmetrical and uncontaminated by outliers, a standard test such as the paired t-test will be used; if this test is compromised by outliers or an extremely non-normal distribution, then a rank-based alternative will be used. While this study has not been powered for this comparison, in the absence of any information regarding the variability of this response, this will enable an estimate to be made of the variability, so that future studies may be based on a reasonable assessment of power.

## Analysis of Demographics

Demographic characteristics (age, sex, race) of each study cohort will be tabulated.

## Analysis of Safety

The overall percentage of subjects reporting solicited and unsolicited AEs will be tabulated by type of event, by severity and by relationship to the investigational product.

## Interim Analysis

No interim analyses will be conducted during the study.

# HUMAN SUBJECTS PROTECTION

## Regulatory Considerations

Starpharma or its agent will submit the appropriate documents to the local regulatory agencies and IEC/IRBs and will await approval prior to study commencement.

This study will be conducted in accordance with the following guidelines and regulations:

- International Conference on Harmonization (ICH) Guidelines for Good Clinical Practice (GCP) (E6)
- ICH Guidelines for Good Clinical Practice annotated with TGA comments (July 2000)
- NHMRC “National Statement on Ethical Conduct in Research Involving Humans”
- The Declaration of Helsinki
- US FDA Human Subject Protection Regulations (Title 21 Code of Federal Regulations (CFR), Parts 50, 54, 56 & 312, and Title 45 CFR Part 46)
- NIAID Clinical Terms of Award (<http://www.niaid.nih.gov/ncn/pdf/clinterm.pdf>), and other guidance (<http://grants.nih.gov/grants/guide/notice-files/NOT-OD-00-039.html>, and <http://grants.nih.gov/grants/guide/notice-files/NOT-OD-00-038.html>).

## Independent Ethics Committee (IEC)/Institutional Review Board (IRB)

Prior to the commencement of the clinical study, written IEC/IRB approval from the Alfred Hospital Ethics Committee must be received by the Investigator. Subject recruitment will not start until satisfactory evidence of ethical approval is given by the Investigator to Starpharma in writing. Starpharma requires written approval that clearly identifies the study protocol by title, number, and version date. The study site must have copies of the IRB procedures and roster (or a statement of appropriate constitution of the IRB) in their Study File.

## Interpretation of the Protocol / Protocol Amendments

With the exception of emergency situations, no changes or deviations in the conduct of the signed protocol will be permitted without the prior documented approval of Starpharma, the Starpharma Medical Monitor/responsible physician, DAIDS Medical Officer and the IEC/IRB. In the event of an emergency, the Investigator will institute any medical procedures deemed appropriate. However, all such procedures must be promptly reported to Starpharma, the Starpharma Medical Monitor/responsible physician and the IEC/IRB.

Administrative changes of the protocol are defined as minor corrections and/or clarifications that have no effect on the way the study is to be conducted. These administrative changes will be agreed upon by Starpharma, the DAIDS Medical Officer and the investigator and will be documented in a memorandum. The IEC/IRB will be notified of administrative changes by the investigator. Starpharma will submit the clarification memo to the DAIDS Medical Officer.

Following approval of protocol amendments, an amended final protocol will be prepared.

## Subject Informed Consent

Written informed consent will be obtained from all potential study participants prior to the initiation of any study-related procedures. In obtaining and documenting informed consent, the investigators will comply with applicable local and domestic regulatory requirements and will adhere to GCP and to the ethical principles that have their origin in the Declaration of Helsinki.

Prior to the beginning of the trial, site investigators will have the IRB/EC’s written approval/favourable opinion of the protocol, informed consent forms, and any other study-related information to be provided to participants. This study does not plan to enrol children under 18 or illiterate individuals. All study related materials including the informed consent forms will be available in English.

The informed consent process will give individuals all of the relevant information they need in order to decide whether to participate, or to continue participation, in this study. Potential research participants will be permitted to ask questions and to exchange information freely with the study investigators. Only listed study investigators may obtain informed consent from potential study participants. The investigators will keep research participants fully informed of any new information that could affect their willingness to continue study participation.

## Confidentiality

Members of the study staff sites are all trained in subject confidentiality. The log of study participant names and other protected health information will be kept in a double-locked area. All computer information about study volunteers will be kept on a computer with log-on passwords. Laboratory specimens are labelled with study numbers and date, and are delivered or shipped by study staff. The study sites’ data management and clinical staff are the only personnel with access to the protected health information of study volunteer. Each member of the staff has a log-on identification and password, logs off before leaving a computer screen unattended, and closes their office door when out of the office. All research records will be kept indefinitely following closure of the study.

Starpharma will preserve the confidentiality of subjects taking part in this study. In the event of names inadvertently appearing on study documentation, this information will not be processed.

Subject medical records pertaining to this study may be inspected/audited at any time by Starpharma employees or their duly authorised representatives, a regulatory authority or the IEC/IRB. All records accessed will be strictly confidential. Consent to participate in this study includes consent to these inspections/audits.

# ADMINISTRATIVE ASPECTS

## Clinical Trial Agreement

Prior to commencement of the study, the Investigator must sign a clinical trial agreement that will clearly delineate the responsibilities and obligations of investigator and sponsor and will form the contractual basis under which the clinical trial will be conducted.

A Clinical Trial Agreement will also be put in place between DAIDS and Starpharma.

## Study File

All associated study correspondence will be filed by the Investigator and will be available for inspection by the Sponsor or designee to determine that all documentation is present. It will be responsibility of the Investigator to provide adequate means for organisation and filing of study documentation at the study centres.

## Initiation of the Study

Prior to the commencement of the study, designated representatives from Starpharma and DAIDS will visit the investigational site to ensure adequacy of facilities and to discuss with the Investigator, and other personnel involved with the study, their responsibilities with regard to protocol adherence.

The investigational staff may not enrol any subjects prior to receipt of written approval from the IEC by Starpharma, and completion of a formal meeting conducted by the Starpharma representative to initiate the study. This meeting will include an inventory of study supplies and a detailed review of the Protocol and CRFs. In addition, study participants may not be enrolled until the site has been activated by DAIDS and written confirmation of this activation has been received by Starpharma.

## Subject Reimbursement

Subjects will be reimbursed according to the guidelines of the IEC/IRB in order to compensate them for their inconvenience and time.

## Subject Identification

All subjects screened for a study will be allocated a unique screening identifier and entered chronologically on the Subject Log at the initial visit. In the event that a subject is not included in the study, the reason is to be documented in the space provided on the Subject Log.

The eligible subjects entering the Study will be assigned a Subject Allocation/Randomisation Number in sequential order. The Subject Allocation/Randomisation Number will be entered on all pages of the CRF.

Although pre-study samples will be couriered directly from the study participant’s home, the courier will transport the sample concealed in an opaque bag, and be unaware of the nature of the sample (except that it is biological in nature and un-infected), the study for which it is used, and any of the subject details. The opaque bag will contain the participant and sample identification numbers, will be sealed and tamper evident to prevent inspection by the courier.

## Confidential Follow-up

The investigator will be responsible for retaining sufficient information about each subject (e.g. name, address, phone number, and identity in the study) so that regulatory agencies or Starpharma may access this information should the need to do so arise. These records should be retained in a confidential manner, as legally mandated according to local requirements, and stored separately from all other study documentation.

## Recording of Data

The investigator should maintain the individual subject files separate to the CRFs. The files should include visit dates of the subject, records of vital signs, medical history or examinations administered, laboratory results, concomitant treatments. Any AE encountered and other notes as appropriate. This constitutes ‘source data’. All entries on the CRFs must be backed up by source data, unless agreed that the CRF will constitute source data.

The CRFs must be kept in order and up-to-date so that they always reflect the latest observations on the subjects enrolled in the study.

Each subject’s file should have attached to it the original signed Informed Consent. When the study treatment is completed, the Informed Consent should be kept on file with a copy of the completed CRF in the appropriate file folder provided, or a note made indicating where the records can be located. All records should be kept in conformance with applicable national laws and regulations.

All original laboratory reports should be available for review in each subject’s file. It is important that the original reports are available for review because of the possibility of inaccuracies or errors in transcribing data from original records to the CRF.

CRFs must be completed legibly for each subject enrolled in the study and signed-off by the investigator. This should be done as soon as possible after completion of therapy.

## Monitoring of the Study

The investigator will permit Starpharma and its agents to monitor the study as frequently as Starpharma deems necessary to determine that data recording and protocol adherence are satisfactory. An independent Study Monitor will collect CRFs and to verify subject data on the CRFs for analysis.

The investigator will allow Starpharma and its agents reasonable access to the CRF and related source documents for monitoring purposes as frequently as Starpharma deems necessary. This includes tests performed as a requirement of participation in this study and may also include other medical records required to confirm information contained in the CRF such as past history and secondary diagnoses.

At each subject visit, the investigator or delegate should record all data generated since the last visit on the CRF. The investigator and his/her staff will be expected to co-operate with the monitor to assist in providing any missing information.

The Study Monitor will require access to the Investigator’s study file to ensure completeness of all study-related documentation. The Study Monitor will provide the investigator with adequate means for organization and filing of study documentation at the study centre.

The date the Study Monitor visits the investigational site will be recorded in the site visit log. During monitoring visits, the study site co-ordinator and investigator should be available, the source documentation will be accessible and a suitable environment will be provided for the Study Monitor to review study related documentation.

The key purposes for monitoring visits by the Study Monitor include the following:

- 1. Helping to resolve any problems.
  2. Examining all study documentation for completion, adherence to the protocol and possible adverse events.
  3. Discussing inconsistencies or missing data.
  4. Ensuring all study materials are correctly stored and dispensed.
  5. Verification of study data with source documents.
  6. Checking fulfilment of the obligations of the Investigator.
  7. Reviewing consent forms and date of consent.
  8. Inspecting study drug (storage, labelling and documentation).

The investigator will provide Starpharma with the completed and signed CRFs at the end of the study period planned for each subject within 4 weeks of the last subject completing the study.

## Protocol Deviations

Study subjects who are found not to have complied with the requirements of the protocol must be reported to Starpharma, and subsequently by Starpharma to the DAIDS Medical Officer. Such subjects may be withdrawn from the study or statistical analyses at the discretion of the Investigator/Starpharma.

Deviations from the protocol should not be made other than as part of a protocol amendment agreed with Starpharma and DAIDS, except where necessary to eliminate an immediate hazard to study subjects. If a protocol deviation occurs, the Investigator must notify Starpharma and the appropriate IEC/IRB as soon as possible. Starpharma must notify the DAIDS Medical Officer.

Evaluability of such subjects for analysis will be decided through discussion between Starpharma, the study statistician, the investigator and the DAIDS Medical Officer at the end of the study. All protocol deviations must be noted and explained in the Investigator’s file.

Protocol deviations can be divided into two categories as follows:

Minor Protocol Deviations

- - all instances where the protocol specified instructions have not been followed during the on-study period. For example, a subject does not provide a blood sample as required or a visit occurs outside the acceptable window period.

Major Protocol Deviations (also known as “Protocol Violations”)

- - when the subject did not meet all inclusion and/or exclusion criteria prior to entry into the study;
  - if additional blood samples are taken or other procedures performed that are not specified in the protocol.

## Data Quality Control

Throughout the Study, the data will be monitored and the CRFs checked against the subject’s medical record for completeness and accuracy. This will be performed by the Study Monitor.

Following completion and collection of the CRFs, the data will be checked manually, entered into a database and electronically checked for consistency and range. Queries will be generated for spurious data and clarification sought from the responsible Investigator or delegate at the Study site.

These data queries must be resolved in a timely manner by the Study site.

All laboratory assays will be conducted according to the protocol as laid out in the Study Procedures Manual and standard laboratory-specific SOPs. The laboratory responsible for generation of primary endpoint data will be subject to audit during the course of the study to ensure compliance with these procedures.

## Quality Assurance Audit/Inspection

The Study may be subject to an audit by an authorised representative of Starpharma and/or an authorised Regulatory Authority (e.g. TGA, FDA).

Regulatory authorities may request access to all study documentation, including source documents for inspection and copying, in keeping with local regulations. Starpharma will immediately notify the Investigator of an upcoming audit/inspection.

In the event of an audit, all pertinent study-related documentation must be made available.

If an audit or inspection occurs, the Investigator will permit the auditor/inspector direct access to all relevant documents and allocate his/her time as well as the time of relevant staff to discuss the findings and any relevant issues.

## Study and Site Closure

Starpharma reserves the right to prematurely discontinue or suspend the study either at a particular site or at all sites at any time and for any reason. If such action is taken, a Starpharma Clinical Representative will discuss this with the Investigator at that time and notify the Investigator in writing. If the study is suspended or terminated for safety reasons the Investigator conducting the study will be immediately notified of the action as well as the reason for it, as will the relevant regulatory agencies. The Investigator will advise the IEC/IRB overseeing the study at their site.

Upon closure of the study (whether at the expected conclusion or prematurely), the following activities will be checked by the Starpharma Clinical Representative, and the Principal Investigator will be notified of any discrepancies for investigation, correction and/or explanation:

1. Return of all study data
2. Data clarification and resolution of queries
3. Study product/drug accountability, reconciliation and final disposition
4. Review of site study records for completeness
5. Shipment of any relevant samples to the central laboratory

## Record Retention

All study documents, including the protocol and CRFs, are the confidential property of Starpharma and should be regarded as such. Unused CRFs must be returned to Starpharma or destroyed at the end of the study. Completed CRFs will be returned to Starpharma during the study by a method agreed by the study monitor. A study document binder will be provided by Starpharma for all required study documents. A checklist of all records to be retained by the Investigator will be provided by Starpharma.

Following completion of the study, the Investigator will retain copies of the approved protocol, completed CRFs, informed consent documents, relevant source documents, and all other supporting documentation related to the project in accordance with the applicable regulatory requirements. In Australia, documents must be retained for a minimum of 15 years from the date of termination of the study or for at least 2 years after the last approval of a marketing application in an ICH region, or at least 2 years after the formal discontinuation of the clinical development of an investigational product. Starpharma will confer with NIAID/DAIDS to assure that record retention is in accordance with current NIAID/DAIDS policies.

In the event that the Investigator retires or relocates, custody of the records may be transferred to another suitable person who will accept responsibility for the records. Notice of such transfer should be given to Starpharma Pty Limited in writing.

## Study Report

A complete study report and its results shall be written on completion of the study and will include any conclusions drawn with respect to the safety and efficacy of the study product (refer to ICH Topic E3 - Note for Guidance on Structure and Content of Clinical Study Reports (CPMP/ICH/137/95)).

Starpharma may also request the preparation of an interim report for submission to a regulatory agency. Starpharma or its agent will write the report in consultation with a nominated Investigator (or nominee). It is the expectation of Starpharma that the Investigator will sign the final study report.

Progress reports will be provided to the NIH, FDA, IRB/IEC and all other relevant regulatory bodies in accordance with their requirements.

# INDEMNITY AND COMPENSATION

Starpharma will provide compensation to participants for any injury suffered as a result of participation in the study in accordance with the Medicines Australia Guidelines for compensation for injury resulting from participating in a company-sponsored research project. A copy of the Medicines Australia Guidelines is available to participants from the research staff on request.

In the event that participants suffer an injury as a result of participating in this research project, care and treatment will be provided by the public health service at no extra cost to the participant.

## Insurance

Starpharma has taken out insurance to cover its obligations under Indemnity and compensation guidelines for injury to healthy volunteers involved in the Study.

# SPONSOR RESPONSIBILITIES

In addition to preparing the study protocol, the Sponsor or its agent will also be responsible for the conduct of the activities listed below.

## Funding

All direct costs associated with the conduct of the study and laboratory investigations will be paid for by Starpharma as outlined in the Clinical Trial Agreement. Starpharma will be reimbursed by NIAID for the cost of conducting this study.

## Supply of Study Materials and Study Documentation

Starpharma or its agent will supply the study materials including CRFs, subject Diary Cards and other associated documentation required for the study.

## Compliance with Regulatory Requirements

Starpharma will ensure that the Investigator is conducting the study in accordance with the local and international regulatory requirements as stipulated in the protocol.

## Transfer of Sponsor Obligations

Transfer of Sponsor Obligations may occur for certain activities such as monitoring and data management.

# USE OF DATA AND PUBLICATIONS

The principles for publication of Results of this study will be addressed in Clinical Trial Agreements between Starpharma and NIAID, and Starpharma and the subcontractors performing the study.

[Note: Results means any and all information and know how (whether patentable or not) which is discovered, invented or developed or which arises in the course of or as a result of the conduct of the Study, including any and all improvements to the products being studied.]

# APPENDICES

## References

Joint United Nations Programme on HIV/AIDS (UNAIDS) and World Health Organization (WHO). AIDS Epidemic Update. December 2007.

Corey, L, Wald, A, Celum, CL et al. The effects of herpes simplex virus-2 on HIV-1 acquisition and transmission: a review of two overlapping epidemics. *J Acquir Immune Def Syndr.* 2004;35:435–445.

Haynes BF. HIV vaccines: where we are and where we are going. *Lancet.* 1996;348(9032):933-937.

Elias CJ, Heise LL. Challenges for the development of female-controlled vaginal microbicides. *AIDS.* 1994;8(1):1-9.

Elias CJ, Coggins C. Female-Controlled Methods to Prevent Sexual Transmission of HIV. *AIDS.* 1996;10 Suppl 3:S43-S51.

Mauck C, Doncel G. An Update on Vaginal Microbicides. *Curr Infect Dis Rep.* 2001;3(6):561-568.

Mauck C, Rosenberg Z and Van Damme L, for the International Working Group on Microbicides. Recommendations for the Clinical Development of Topical Microbicides: An Update. *AIDS.* 2001;15(7):857-868.

Wald, A. et al. Effect of Condoms on Reducing the Transmission of Herpes Simplex Virus Type 2 from Men to Women. *JAMA.* 2001;285(24):3100-3106.

Frechet JM, Tomalia DA. Dendrimers and Other Dendritic Polymers. Chichester: Willey. 2001

McCarthy TD, Karellas P, Henderson SA, et al. Dendrimers as Drugs: Discovery and preclinical and clinical development of dendrimer-based microbicides for HIV and STI prevention. Molecular Pharmaceuticals 2005:312-8

Starpharma Pty Ltd. Investigators’ Brochure: SPL7013 (VivaGel®) Version CIB-001-06. Starpharma Pty Ltd. 2006.

Witvrouw M. Fikkert V, Pluymers W, et al. Polyanionic (i.e. polysulfonate) dendrimers can inhibit the replication of human immunodeficiency virus by interfering with both virus adsorption and later steps (reverse transcriptase/integrase) in the virus replicative cycle. Mol Pharmacol. 2000 Nov;58(5):1100-8.

Bernstein DI, Stanberry LR, Sacks S, et al. Evaluations of unformulated and formulated dendrimer-based microbicide candidates in mouse and guinea pig models of genital herpes. Antimicrob Agents Chemother 2003 Dec:47(12):3784-8

Bourne N, Stanberry LR, Kern ER, Holan G, Matthews B, Bernetein DI. Dendrimers, a new class of candidate topical microbicides with activity against herpes simplex virus infection. Antimicrob Agents Chemother 2000 Sep:44(9):2471-4.

Jiang YH, Emau P, Cairns JS, et al. SPL7013 gel as a topical microbicide for prevention of vaginal transmission of SHIV89.6P in macaques. AIDS Res Hum Retroviruses 2005 Mar;21(3):207-13.

Dezzutti CS, James VN, Ramos A, et al. In vitro comparison of topical microbicides for prevention of human immunodeficiency virus type 1 transmission. Antimicrob Agents Chemother 2004 Oct;48(10):3834-44.

Cummins J, Abner SR, Morken T, et al. Human cervical and colorectal explants for safety and efficacy testing of topical microbicides. Microbicides 2004, London UK 2004.

Abner SR, Guenthner PC, Guarner J, et al. A Human Colorectal Explant Culture to Evaluate Topical Microbicides for the Prevention of HIV Infection. J Infect Dis 2005 Nov 1;192(9):1545-56.

Patton DL, Cosgrove Sweeny YT, McCarthy TD, Hillier SL. Preclinical safety and efficacy assessments of dendrimer-based (SPL7013) microbicide gel formulations in a nonhuman primate model. Antimicrob Agents Chemother 2006 May;50(5):1696-700.

Clarke J, et al Microflora Changes with the use of a vaginal microbicide. Sex. Trans. Dis 2002 29(5) 288-293

Flynn A et al. Volumetric self-sampling of cervicovaginal fluid to determine potential fertility: a multicentre pre-effectiveness study of the Rovumeter. *Human Reproduction* 1997;12(8):1826-1831

Pratt D. et al. Correlation of cervicovaginal fluid volume with serum estradiol levels and total follicular volume during human gonadotropin stimulation. *J. Assist. Reprod. Genet.* 1992;9(1):14-8

Barnhart. K et al. Distribution of a 3.5mL (1.0%) C31G vaginal gel using magnetic resonance imaging. *Contraception* 2005;71:357-61

Boskey et al, A Self-Sampling Method to Obtain Large Volumes of Undiluted Cervicovaginal Secretions; Sexually Transmitted Diseases; Feb. 2003, Vol. 30, No. 2 p107-109

Brache, V, Cohen, JA, Cochon, L, Alvarez, F. Evaluating the clinical safety of three vaginal applicators: a pilot study conducted in the Dominican Republic. *Contraception.* 2006;73:72-77.

Klasse PJ, Shattock RJ and Moore JP. Which topical microbicides for blocking HIV-1 transmission will work in the real world? *PLoS Med*. 2006;3(9):e351

Shattock RJ and Moore JP (2003) Inhibiting sexual transmission of HIV-1 infection. *Nature Reviews Microbiology* 1: 25 – 34.

Wei X, Decker JM, Liu H, Zhang Z, Arani RB et al (2002) Emergence of resistant human immunodeficiency virus type I in patients receiving fusion inhibitor (T-20) monotherapy. *Antimicrob Agents Chemother* 46: 1896 – 1905.

Waping J, Moore KL, Sonza S, Mak J, Tachedjian G (2005) Mutations that abrogate human immunodeficiency virus type I reverse transcriptase dimerization affect maturation of the reverse transcripase heterodimer. *Journal of Virology* 79: 10247 – 10257.

Sudo K, Konno K, Yokoto, T and Shigeta S (1994). A sensitive assay system screening antiviral compounds against herpes simplex virus type 1 and type 2. *Journal of Virological Methods* 49: 169 – 178.

## Declaration of Helsinki

The Declaration of Helsinki can be found at the following link:

<http://www.wma.net/e/policy/b3.htm>

## Outcomes, Diagnostics and Follow-Up Evaluations

| **Condition** | **Study gel Use** | **Evaluation** | **Follow up and Treatment Action** |
| --- | --- | --- | --- |
| Deep Epithelial Disruption (Ulceration) | Hold study Gel (until evaluated) | Swab for herpes simplex culture.  Perform syphilis serology (Herpes serology optional) | Re-evaluate in 48-72 hours and reinstate study gel use if resolved. If the ulcer has become worse or not healed in 48-72 hours follow the lesion per local standard of care. Ask participant to return in 7-10 days for follow up syphilis serology. If there is reoccurrence and there is no other aetiology, then consider permanent discontinuation. |
| Superficial Epithelial Disruption (Abrasion/Peeling) | Continue | Naked eye evaluation | Re-evaluate by speculum examination in 48-72 hours. If condition is significantly worse, hold study gel use. Otherwise continue study gel use. |
| Localised erythema or oedema: area of less than 50% of vulvar surface or combined vaginal and cervical surface | Continue | Naked eye evaluation | If asymptomatic, re-evaluate at next regularly scheduled visit. If symptomatic, re-evaluate by speculum examination in 5-7 days. If worsened significantly, hold study gel use, until further evaluation is scheduled. Otherwise, continue study gel use. |
| Generalised erythema or severe oedema; area of more than 50% of vulvar surface or combined vaginal and cervical surface affected by erythema | Hold study Gel (until evaluated) | Naked eye evaluation | Re-evaluate in 48-72 hours and reinstate study gel use if resolved. If there is reoccurrence and there is no other aetiology, then consider permanent discontinuation. |
| Abnormal vaginal discharge (not considered due to gel leakage) | Hold study gel (until evaluated) | Perform wet mount for Candida vaginitis, trichomoniasis, and BV | Provide treatment for all cases of trichomoniasis, symptomatic Candida vaginitis, and symptomatic bacterial vaginosis. Gel use to be paused until 5 days after both cessation of treatment and symptoms for all cases of symptomatic Candida vaginitis, and symptomatic bacterial vaginosis. Gel permanently discontinued and subject withdrawn in cases of trichomoniasis. Gel use may be continued without treatment in the presence of asymptomatic Candida vaginitis and/or asymptomatic bacterial vaginosis |
| Unexpected genital bleeding | Continue (at clinician’s discretion) | Naked eye evaluation | If determined to be due to deep epithelial disruption, refer to guidelines in this table. Otherwise continue gel use. |
| Presumed cervicitis (findings on exam such as mucopurulent cervical discharge) | Hold study gel (until evaluated) | Evaluate for N. gonorrhoeae and C. trachomatis | Provide treatment and permanently discontinue gel use / withdraw participant for all cases of cervicitis. |
| EAE that is judged by the site investigator or designee to be definitely, probably, possibly, or probably not related to the study gel or applicator | For Grades 1,2, and 3 – Hold study Gel (until evaluated)  For Grade 4 – Permanent Discontinuation / withdraw participant | Evaluate as according to current clinical practice at the site  Not applicable | Provide treatment as clinically indicated, when resolved reinstate study gel use at clinician’s discretion.  Not applicable |

1. The 0 hour sample will be taken between 2–10 minutes after dose application. [↑](#footnote-ref-2)
2. Minimum 5 day wash-out between Treatment periods. [↑](#footnote-ref-3)
3. Three separate CV samples will be taken on different days at a minimum 24 hours between samples [↑](#footnote-ref-4)
4. Hepatitis B antigen, anti-HCV antibodies, anti-HIV antibodies with associated information and counselling [↑](#footnote-ref-5)
5. Pap smear performed unless documented result (within 12 months) available [↑](#footnote-ref-6)
6. Including pelvic examination [↑](#footnote-ref-7)
7. If necessary, the study participant may stay overnight at the clinical unit. [↑](#footnote-ref-8)
8. AEs reported pre-first study dose will be recorded as “screening/baseline signs and symptoms” [↑](#footnote-ref-9)
